# Genome-Wide Identification and Analysis of BAHD Acyltransferases Involved in Anthocyanin Biosynthesis in *Perilla frutescens*

**DOI:** 10.3390/biology15110859

**Published:** 2026-05-30

**Authors:** Peina Zhou, Bingyan Gao, Chenghao Fei, Jiangqiong Luo, Ling Gong

**Affiliations:** 1 College of Pharmacy, Hubei University of Chinese Medicine, Wuhan 430065, China; zhoupeina@163.com; 2 Nanjing Research Institute for Comprehensive Utilization of Wild Plants, Nanjing 210042, China; 15738237785@163.com; 3 Institute of Chinese Medicinal Materials, Nanjing Agricultural University, Nanjing 210095, China; feichenghao@njau.edu.cn; 4 Chongqing Academy of Chinese Materia Medica, Chongqing 400065, China; 5 Hubei Shizhen Laboratory, Wuhan 430065, China

**Keywords:** *Perilla frutescens*, BAHD acyltransferases, anthocyanin, gene expression, molecular docking

## Abstract

*Perilla frutescens* (L.) Britt., a medicinal and edible herb, is valued for its diverse leaf coloration, attributed to varying levels of anthocyanin accumulation. The primary anthocyanins in *P. frutescens* are acylated cyanidin glycosides; however, the enzymes facilitating the acylation process have yet to be characterized. BAHD acyltransferases, known to catalyze such modifications, remain uncharacterized in *P. frutescens.* To systematically identify potential genes that may be associated with this function, we performed a comprehensive genome-wide analysis of the BAHD acyltransferase family in *P. frutescens*. Our study identified 134 *PfBAHD* genes, which were subsequently analyzed for their physicochemical properties, phylogenetic relationships, conserved domains, motif compositions, and promoter cis-elements. Phylogenetic analysis categorized the *PfBAHD* genes into six clades, with Clade I being the primary candidate for anthocyanin-related activity due to its enrichment with members known to acylate flavonoids in other species. Promoter analysis indicated a significant presence of cis-elements associated with light, phytohormones, and stress responses. By integrating tissue-specific metabolomic and transcriptomic data, we established correlations between anthocyanin accumulation patterns and *PfBAHD* gene expression. Through the integration of multi-omics data, six candidate genes were prioritized, with *PfBAHD05*, *PfBAHD77*, and *PfBAHD112* emerging as the most promising candidates. These genes demonstrated predominant expression in leaves, were induced under conditions of high light exposure, and were predicted to be localized in the cytoplasm. To further explore their potential functions, molecular docking analyses were conducted, suggesting that PfBAHD77 may have a preference for highly glycosylated anthocyanins, whereas PfBAHD05 and PfBAHD112 may favor substrates with lower levels of glycosylation. Collectively, these findings provide a preliminary foundation for understanding anthocyanin acylation in *P. frutescens* and identify several BAHD candidate genes that could be potentially targeted in future metabolic engineering efforts pending further biochemical and genetic validation.

## Simple Summary

*Perilla frutescens* is a medicinal and culinary herb with leaves containing acylated anthocyanins, which are stable red pigments. The enzymes that add acyl groups to these anthocyanins have not been identified in perilla. In this study, we searched the perilla genome and found 134 BAHD acyltransferase genes, a family known for such modifications in other plants. By analyzing gene expression together with pigment data, we selected three candidate genes—*PfBAHD05*, *PfBAHD77*, and *PfBAHD112*—that are expressed mainly in leaves and change with light conditions. Molecular docking results suggested that PfBAHD77 may prefer more complex anthocyanins, whereas PfBAHD05 and PfBAHD112 may favor simpler ones. These findings provide information on possible genes involved in anthocyanin acylation in *P. frutescens* and may be useful for future studies on natural pigment production.

## 1. Introduction

*Perilla frutescens* (L.) Britt., a species within the Lamiaceae family, represents an economically significant herb extensively cultivated across East Asia. In addition to its traditional applications in medicine and cuisine, it is valued for its production of unique natural pigments [1,2,3]. This property is conferred by its synthesis of distinctive acyl-modified anthocyanins, predominantly shisonin and malonylshisonin. These compounds demonstrate enhanced stability and bioactivity compared to conventional anthocyanins, rendering them promising candidates for use in nutraceuticals and as natural colorants [4]. Nonetheless, their broader industrial application is impeded by an incomplete understanding of their biosynthetic pathways, particularly the final acylation step, which is vital for their unique chemical structures and properties.

The biosynthesis of shisonin and malonylshisonin originates from cyanidin through a series of glycosylation and acylation steps [2,5,6]. Although numerous upstream enzymes have been identified, the specific anthocyanin acyltransferases responsible for the characteristic coumaroyl and malonyl modifications have yet to be determined. Drawing upon the conserved acylated structures and established biochemical pathways in other species, it is strongly suggested that the enzyme responsible is a member of the BAHD acyltransferase superfamily using p-coumaroyl-CoA and malonyl-CoA as acyl donors [7]. This superfamily is characterized by conserved HXXXD and DFGWG motifs and is phylogenetically categorized into six clades, each with distinct functional specializations [8,9]. In particular, members of Clade I predominantly catalyze the acylation of secondary metabolites, including flavonoids, anthocyanins, and phenolic glycosides [10]. For example, enzymes involved in the acylation of anthocyanins in various species, such as *Dahlia variabilis*, *Salvia splendens*, and *Arabidopsis thaliana*, consistently group within Clade I. Clade II is associated with the biosynthesis and elongation of cuticular waxes [11]. Clade III comprises a series of alcohol acyltransferases responsible for the synthesis ofvolatile esters, which contribute to the aroma of flowers and mature fruits and can function in plant defense, such as acylsugars from *Solanum* trichomes [12,13]. Clade IV primarily includes agmatine coumaroyltransferases (ACTs) [14,15,16]. Members of Clade V predominantly exhibit hydroxycinnamoyl transferase (HCT) activity and are involved in the synthesis of polymers such as lignin [17]. In contrast, enzymes within Clade VI demonstrate a broader substrate spectrum and functional diversity, utilizing substrates ranging from terpenoids to medium-chain alcohols [18]. Nonetheless, the comprehensive systematic identification, molecular characteristics, and evolutionary relationships of the BAHD acyltransferase family in *P. frutescens* remain entirely unknown. The existing gap poses a significant challenge to the optimization and engineering of the production of these valuable compounds.

In response, this study conducted a comprehensive genome-wide analysis to identify and characterize the *PfBAHD* genes in *P. frutescens*. We analyzed their sequence features, phylogenetic relationships, conserved motifs, gene structures, promoter cis-elements, and chromosomal locations [19]. Given that anthocyanins predominantly accumulate in leaves, we examined the correlation between tissue-specific (leaf, stem, root) anthocyanin and flavonoid profiles and *PfBAHD* expression to prioritize candidate genes. This multi-omics integration, alongside expression analysis, highlighted three potential key candidates: *PfBAHD05*, *PfBAHD77*, and *PfBAHD112*. Subsequent analyses, including predicted subcellular localization and molecular docking, provided preliminary insights into their possible roles in anthocyanin acylation. Collectively, this research may serve as a preliminary genetic foundation for future metabolic engineering efforts aimed at optimizing anthocyanin production in *P. frutescens*, pending further experimental validation.

## 2. Materials and Methods

### 2.1. Identification of BAHD Family Genes in P. frutescens

The genome sequence of *P. frutescens* was obtained from the National Genomics Data Center (NGDC; accession: PRJNA431002) [19]. BAHD family members were identified by scanning the genome with the Pfam HMM profile for BAHD (PF02458) using TBtools [20]. Candidate sequences were verified by CDD and SMART to confirm intact BAHD domains. ProtParam (ExPASy) was used to compute isoelectric point, molecular weight, instability index, and amino acid composition, while subcellular localization was predicted via WoLFPSORT [21].

### 2.2. Phylogenetic Analysis

The amino acid sequences of functionally characterized BAHD acyltransferases from various plant species were retrieved from the NCBI database (https://www.ncbi.nlm.nih.gov/). Multiple sequence alignment was performed using the MUSCLE algorithm implemented in MEGA-X, with default parameters. A maximum-likelihood (ML) phylogenetic tree was constructed with 1000 bootstrap replicates, a partial deletion parameter applied to sites with less than 80% coverage, and the JTT + G + I amino acid substitution model. The resulting tree file (Newick format) was then imported into the ITOL (v7) online software system for visualization (https://itol.embl.de/) [22,23]. Colored outer rings were added to the tree in iTOL to reflect the established functional classification of BAHD proteins, grouping them into distinct clades based on their characterized biochemical activities in other species.

### 2.3. Conserved Motifs, Gene Structures, and Upstream Cis-Acting Elements of PfBAHDs

The PfBAHDs were analyzed for conserved motifs, domains, and gene structures as described [24]. Chromosomal locations, intra-genomic synteny, and promoter cis-elements (2 kb upstream) were determined using TBtools II [25] and PlantCare [26].

### 2.4. Preparation of Plant Materials

Seeds of *P. frutescens* (L.) Britt., sourced from a medicine market in Anguo City (Hebei, China), were stored at 4 °C in our laboratory. Plants were raised in a greenhouse phytotron under controlled conditions (16/8 h light/dark cycle, 25 °C, 60% RH) After three months of growth, leaves, stems, and roots were collected separately. The obtained plant tissues were immediately flash-frozen in liquid nitrogen and stored at −80 °C for subsequent experiments and metabolite analyses. The treatment of different light intensities was carried out in accordance with the reference [27]. In detail, one-month-old *P. Frutescens* seedlings were divided into three groups and exposed to different intensity of light (Group High, 30,000 lx), normal light (Group Middle, 12,000 lx), and low-intensity light (Group Low, 5000 lx) treatments. After five weeks of treatment, leaves were collected as the experimental material. The plant material, *Perilla frutescens* (L.) Britt., was authenticated based on its typical morphological characteristics by Prof. Gong Ling (Hubei University of Chinese Medicine).

### 2.5. Specimen Preparation and Determination of Total Flavonoids and Anthocyanins

The sample leaves were washed with clean water, and then dried in a 37 °C oven to a constant weight. Total flavonoid content was determined using the NaNO_2_-Al(NO_3_)_3_-NaOH method [28]. Dried leaf samples (0.2 g) were extracted with 5 mL of 70% ethanol by sonication at 60 °C for 30 min, followed by standing for 30 min. After centrifugation at 12,000 rpm for 10 min, the supernatant was collected, the residue was re-extracted once, and the combined supernatants were diluted to 10 mL with 70% ethanol. An aliquot (1 mL) was mixed sequentially with 0.3 mL of 5% NaNO_2_ (stand 6 min), 0.3 mL of 10% Al(NO_3_)_3_ (stand 6 min), and 4 mL of 4% NaOH, then diluted to 10 mL with 70% ethanol. The absorbance of the reaction solution was measured at 510 nm (200 µL per well in a 96-well plate), and the total flavonoid content was calculated using the standard curve.

Total anthocyanins were extracted and analyzed based on a previous method [29] with minor modifications. Dried tissue samples (37 °C) were ground into powder. Accurately weighed 0.1 g of powder was mixed with 10 mL of 0.05% HCl-methanol in a centrifuge tube (six biological replicates per group). The mixture was ultrasonicated at 4 °C for 30 min and centrifuged at 12,000 rpm for 10 min. The supernatant was collected and diluted by mixing 60 µL of extract with 940 µL of either pH 1.0 buffer (0.25 M KCl-HCl) or pH 4.5 buffer (0.4 M NaAc-HAc). After 30 min of dark incubation, absorbance was measured at 510 nm and 690 nm. The absorbance of the diluted samples was calculated according to the formula (ΔA): ΔA = (A_510_ − A_690)pH1.0_ − (A_510_ − A_690_)_pH4.5_. According to the formula to calculate the total anthocyanin content, MW = 449.2 g/mol, DF = 1000/60 = 50/3, ε = 26,900 L/(mol·cm), L = 1 cm. Finally, convert the total anthocyanin unit mg/L to mg/g based on the extraction volume unit.

$$\mathrm{Total}\;\mathrm{anthocyanin}\;\mathrm{content}\;(\mathrm{mg}/L)=\frac{\Delta A\times MW\times DF\times 1000}{\varepsilon \times L\times W}$$

### 2.6. The Non-Targeted Metabolomics Analysis

Fresh tissue samples (approx. 100 mg) were homogenized in 400 µL of 80% methanol, followed by ultrasonic-assisted extraction at 4 °C for 30 min. After centrifugation at 13,000 rpm, the supernatant was collected for LC-MS analysis. A quality control (QC) sample was prepared by pooling 20 µL of supernatant from each individual sample [30]. Metabolite profiling was performed on a triple TOF 6600 system (AB SCIEX, Framingham, MA, USA) coupled to a UPLC system (Shimadzu 30A, Shimadzu Corporation, Kyoto, Japan). A 5 µL aliquot was injected onto an Agilent Extend-C18 column (100 mm × 2.1 mm, 1.8 µm) maintained at 40 °C. The mobile phase consisted of (A) 0.1% formic acid and 2% acetonitrile in water, and (B) acetonitrile, at a flow rate of 0.4 mL/min. The gradient elution program was: 0–0.5 min, 2% B; 0.5–7.5 min, 2–35% B; 7.5–13 min, 35–95% B; 13–14.4 min, 95% B; 14.4–14.5 min, 95–2% B; 14.5–16 min, 2% B. Mass spectrometer parameters and compound identification were the same as previously reported [31].

### 2.7. Expression Analysis of PfBAHDs

Transcriptomic data for *P. frutescens* were obtained from the transcriptomes of leaves, stems, and roots (NCBI BioProject: PRJNA690131), with raw reads processed through genome-guided annotation to create gene expression matrices. Standard protocols were used for extracting RNA and synthesizing cDNA from leaves under different lighting conditions or from leaves, stems, and roots [27]. Candidate gene-specific primers for quantitative real-time PCR (qRT-PCR) were designed using NCBI’s Primer-BLAST (Primer3 2.5.0) (https://www.ncbi.nlm.nih.gov/tools/primer-blast/, accessed on 10 May 2025). The amplifications were conducted using an ABI7500 Real-Time PCR System (Applied Biosystems, Foster City, CA, USA) with four technical replicates for each sample, adhering to the manufacturer’s instructions. Table S1 contains the primer sequences. The 2^−ΔΔCT^ method was used to determine relative gene expression levels. Expression patterns were visualized using GraphPad Prism 10.

### 2.8. Molecular Docking

The three-dimensional structures of the substrates were converted into SDF format using Chemdraw 3D software (version 21.0) (PerkinElmer Inc., Waltham, MA, USA). The protein three-dimensional structures (in PDB format) were obtained via the online tool SWISS-MODEL (https://swissmodel.expasy.org/interactive, accessed on 15 May 2025). Molecular docking and binding affinity calculations were performed using the CB-DOCK2 online server (phttp://clab.labshare.cn:10380/cb-dock2/php/index.php, accessed on 15 May 2025) [32,33]. The optimal binding poses for each protein–ligand complex were visualized using PyMOL 3.1.4 software (Schrödinger LLC, New York, NY, USA).

### 2.9. Subcellular Localization of the PfBAHDs

To determine the subcellular localization of PfBAHD05, PfBAHD77, and PfBAHD112, C-terminal GFP fusion constructs were generated. The coding sequences were amplified using primers listed in Table S1 and subsequently cloned into the pCNG-GFP vector, yielding the recombinant expression vectors pCNG-*PfBAHD05*, pCNG-*PfBAHD77*, and pCNG-*PfBAHD112*. These constructs were transformed into *Agrobacterium tumefaciens* strain GV3101 and then infiltrated into leaves of 4-week-old *Nicotiana benthamiana* plants using agroinfiltration [34]. After incubation in darkness for 48 h, the infiltrated leaf sections were excised, mounted in water, and visualized using a laser scanning confocal microscope (Zeiss LSM900, Carl Zeiss AG, Jena, Germany). Empty pCNG-GFP vector was used as a control.

### 2.10. Statistical Analysis

The total flavonoid and anthocyanin content data are presented as the mean ± standard deviation (SD) from six separate biological replicates. Data from RNA-seq, metabolomics, and qRT-PCR were obtained from three separate biological replicates. Statistical analysis was performed using GraphPad Prism 10 (GraphPad Software, San Diego, CA, USA). One-way ANOVA was used for group comparisons, followed by Duncan’s multiple range test, with statistical significance set at *p* < 0.05.

## 3. Results

### 3.1. Identification of and Characterization of the PfBAHDs Acyltransferase Family in P. frutescens

In order to thoroughly identify members of the BAHD acyltransferase family in *P. frutescens*, we conducted a homology search of the genome utilizing a Hidden Markov Model (HMM) profile specific to the BAHD domain (PF02458). Subsequent screening of candidate genes for the conserved HXXXD and DFGWG motifs (Figure S1) resulted in the identification of 134 putative genes, which we have designated as *PfBAHDs*. Notably, the majority of these proteins also contained a YFGN domain. Physicochemical analyses revealed considerable diversity among the PfBAHDs (Table S2). The average protein length was determined to be 443 amino acid residues, with a range spanning from 335 to 791 residues, corresponding to molecular weights between 37.57 and 88.27 kDa. A significant proportion of the proteins (103 out of 134) exhibited an acidic theoretical isoelectric point (pI < 7), were characterized as hydrophilic, and were predicted to be unstable, as indicated by an instability index exceeding 40 in 79 of the proteins. The aliphatic index varied from 72.44 to 98.74. Predictions of subcellular localization suggested that the majority of these proteins are targeted to the cytoplasm (62%) and chloroplast (40%), with a smaller fraction (12%) localized to the nucleus. This observed heterogeneity implies that PfBAHDs may fulfill a variety of functional roles within *P. frutescens*.

### 3.2. Phylogenetic Analysis and Functional Prediction of the PfBAHDs

**Figure 1:**
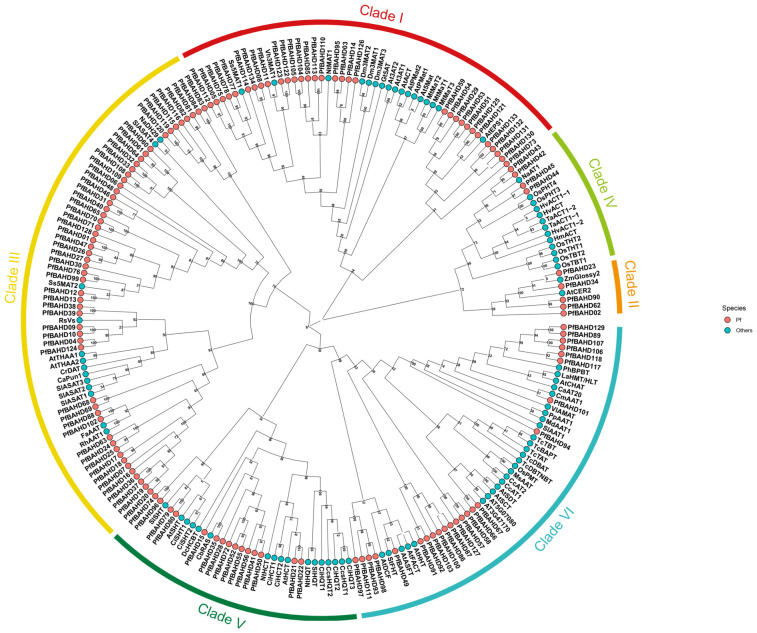
Phylogenetic trees and classification of PfBAHDs and BAHDs of different species. Note: Different background colors indicate different BAHD sub-families.

To clarify the evolutionary relationships of PfBAHDs, a phylogenetic tree was constructed incorporating 134 PfBAHDs alongside functionally characterized BAHD acyltransferases from other species (Figure 1, Table S3). The phylogenetic analysis categorized all proteins into six distinct clades (Clades I-VI), with PfBAHDs distributed across all clades, indicating considerable functional divergence throughout evolution. Clade I comprised 32 PfBAHD members and included functionally characterized enzymes such as Dv3MaT from *Dahlia variabilis*, NtMAT1 from *Nicotiana tabacum*, and At3AT1/At3AT2 from *Arabidopsis thaliana*, which have been documented to participate in anthocyanin acylation [10,35,36]. This suggests that PfBAHDs within this clade may be involved in anthocyanin biosynthesis in *P. frutescens*. In Clade II, included five PfBAHDs (PfBAHD02, 23, 34, 62, and 90), which clustered closely with AtCER2 and its homologs known to mediate the synthesis of epicuticular wax. This implies a potential role for these PfBAHDs in regulating wax accumulation in *P. frutescens*, which may influence the plant’s environmental adaptation and leaf surface properties [37]. Clade III, distinguished as the largest clade (54 members), contained numerous PfBAHDs grouped with enzymes such as RhAAT1 from *Rosa hybrida*, which is involved in the synthesis of volatile esters. This association points to a potential contribution of these PfBAHDs to the biosynthesis of aromatic compounds, thereby influencing the characteristic scent of *P. frutescens* [38]. Clade IV consisted of 4 PfBAHDs, with members like OsPHT3/4 and OsTHT1/2 from *Oryza sativa* primarily facilitating the acylation of various amines. This indicates that the genes from *P. frutescens* within this clade may execute similar functions [39,40]. Clade V (15 members) was further divided into two subclades: SubcladeVa contained four PfBAHDs with homology to spermidine/spermine hydroxycinnamoyltransferases (SHTs) involved in polyamine modification [41]. Subclade Vb are predicted to catalyze acylations using quinic acid as a substrate. Notable examples include NtHCT from *Nicotiana tabacum* and CiHCT2 from *Citrus ichangensis*, which contribute to the biosynthesis of chlorogenic acid and lignin, respectively. This suggests that PfBAHDs in this subclade may play a role in the biosynthesis of phenolic acids in *P. frutescens* [42,43]. Members of Clade VI (24 PfBAHDs) may possess a variety of functions, utilizing substrates ranging from terpenoids to medium-chain alcohols. For example, AtHHT in *Arabidopsis* possesses feruloyl-CoA-dependent feruloyl transferase activity, while AtSCT is considered to be a Spermidine coumaryl-CoA acyltransferase [44,45]. TcBAPT, TcDBAT, and TcDBTNBT from *Taxus cuspidata* are key enzymes in the paclitaxel biosynthesis pathway [46].

### 3.3. Gene Structure and Conserved Motifs Analysis of PfBAHDs

**Figure 2:**
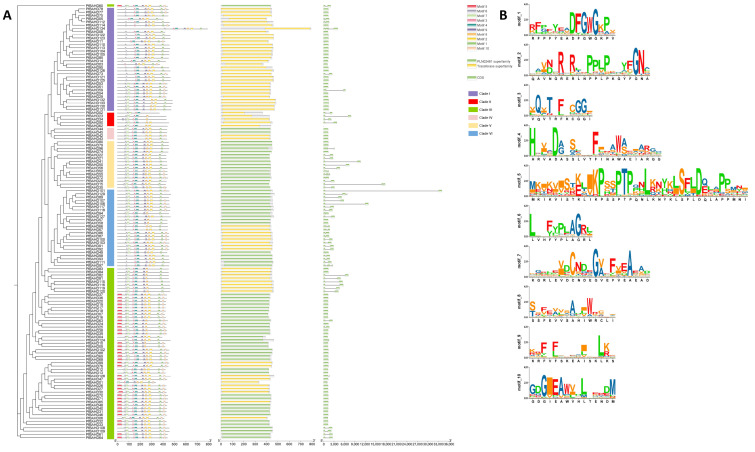
Analysis of conserved motifs, conserved domains and gene structures of PfBAHDs. (**A**). The conserved motifs, conserved domains and gene domains of the PfBAHDs; (**B**). The detail conserved motif sequence of PfBAHDs. Note: In (**A**), rectangles of different colors represent different subfamilies (Clade I–VI) according to the subfamily classification in Figure 1.

The analysis of conserved domains categorized the PfBAHDs into two principal superfamilies: members of Clades I, II, and IV are predominantly associated with the transferase superfamily, whereas those in Clades III, V, and VI primarily belong to the PLN02481 superfamily, both of which are characterized by distinctive acyltransferase domains (Figure 2A). Examination of conserved motifs revealed that motifs 01 (encompassing the DFGWG domain), 02 (YFGN domain), 03, 08, and 09 are ubiquitously present across all family members, underscoring their critical importance. Notably, motif 04, corresponding to the catalytic HXXXD core region, is present in all PfBAHDs with the exception of PfBAHD03. Nevertheless, PfBAHD03 retains the essential HXXXD domain, highlighting the exceptionally high conservation of the HXXXD, DFGWG, and YFGN structural elements, which constitute the fundamental basis for the enzyme family’s catalytic activity (Figure 2B). Interestingly, motif 09 is specific to Clade III but is absent in certain members such as PfBAHD04 and PfBAHD06, suggesting potential functional sub-specialization within this clade. Additionally, an analysis of gene structure demonstrated considerable diversity: 71 out of 134 PfBAHDs lack introns and are primarily concentrated in Clades I, III, and IV. Conversely, genes within Clades II, V, and VI generally possess 1–2 exons, indicating distinct structural patterns among the evolutionary groups.

### 3.4. Cis-Regulatory Element, Chromosomal Location and Duplication Events Analysis of PfBAHDs

**Figure 3:**
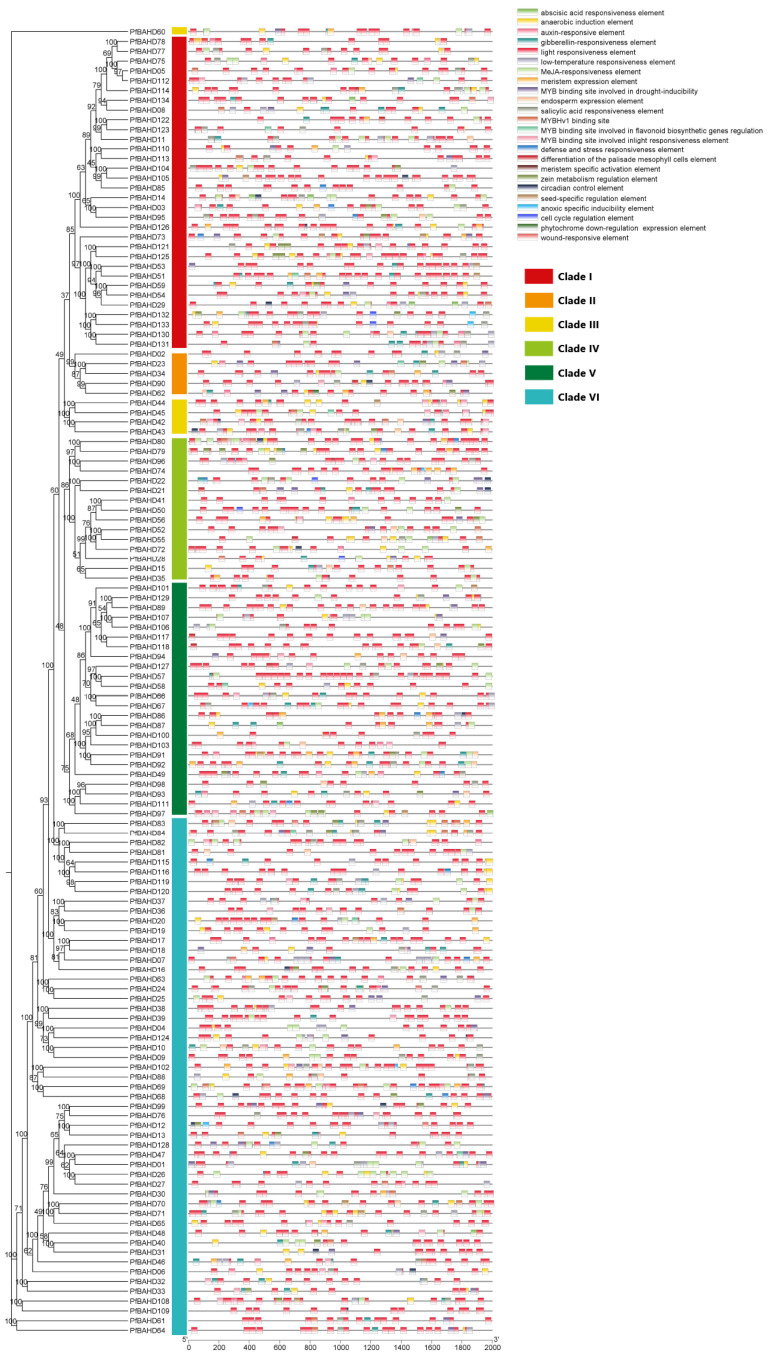
Distribution of cis-regulatory elements into promoter regions of *PfBAHDs*. Note: Different color boxes represent different cis control elements; The rectangles of different colors represent different subfamilies (Clade I–VI) according to the subfamily classification in Figure 1.

The analysis of promoter regions within the 134 *PfBAHDs* revealed the presence of 24 distinct types of conserved cis-regulatory elements (Figure 3). Among these, light-responsive elements were the most prevalent, constituting 52.7% of all identified elements. This was followed by elements associated with phytohormone responses, including those responsive to methyl jasmonate, abscisic acid, gibberellin, and salicylic acid. Additionally, the promoters were enriched with elements responsive to both biotic and abiotic stresses, such as low temperature and drought. These findings suggest that the expression of *PfBAHDs* is likely co-regulated by light signaling, various phytohormones, and environmental stimuli.

**Figure 4:**
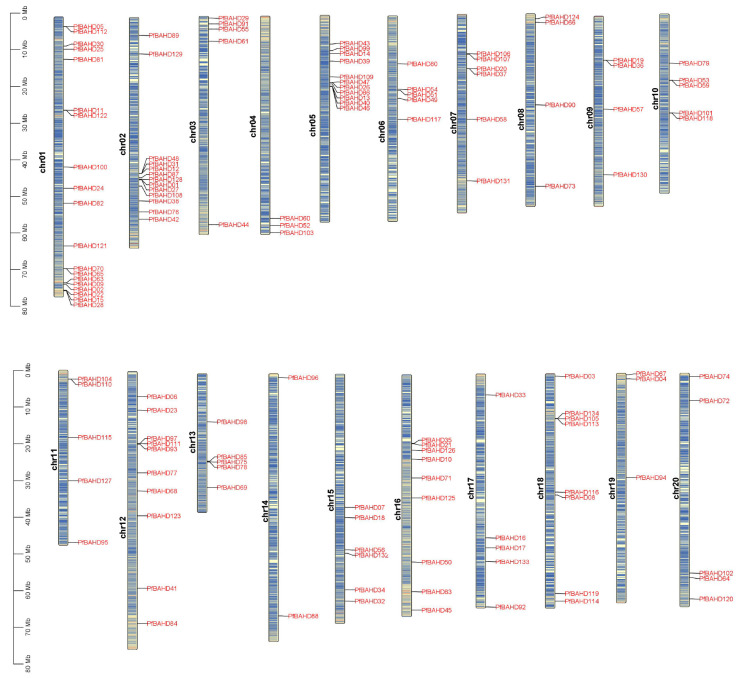
Chromosomal distribution of *PfBAHDs*.

**Figure 5:**
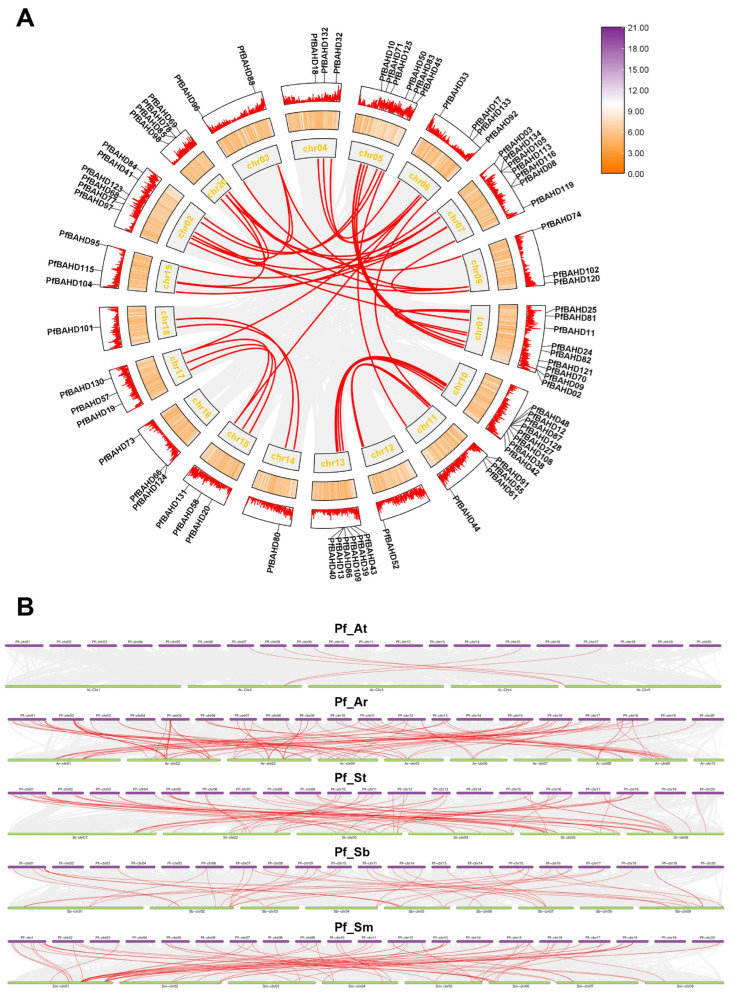
The collinearity of PfBAHDs. (**A**). Intra-genomic collinearity of *PfBAHDs*; (**B**). Collinearity of *PfBAHDs* with the genomes of *P. frutescens* and other species. Note: Pf_At represents the collinearity of *PfBAHDs* between *P. frutescens* and *A. thaliana*; Pf_Ar represents the collinearity of *PfBAHDs* between *P. frutescens* and *A. rugosa*; Pf_St represents the collinearity of *PfBAHDs* between *P. frutescens* and *S. tenuifolia*; Pf_Sb represents the collinearity of *PfBAHDs* between *P. frutescens* and *S. baicalensis*; Pf_Sm represents the collinearity of *PfBAHDs* between *P. frutescens* and *S. miltiorrhiza*.

Chromosomal mapping successfully localized 133 out of the 134 *PfBAHD* genes across 20 chromosomes, with the exception of *PfBAHD62*, which remained unmapped (Figure 4). The distribution of these genes was uneven, with significant clustering observed on chromosomes 1, 2, 4, 12, 16, and 18, while other chromosomes exhibited a more sparse distribution. Collinearity analysis between *P. frutescens* and five related species—*A. thaliana*, *S. tenuifolia*, *S. miltiorrhiza*, *A. rugosa*, and *S. baicalensis*—demonstrated varying degrees of synteny: 5 *PfBAHDs* were collinear with *AtBAHDs*, 51 with *StBAHDs*, 70 with *ArBAHDs*, 55 with *SmBAHDs*, and 35 with *SbBAHDs*. These findings suggest both evolutionary conservation and species-specific diversification (Figure 5A,B).

Within the genome of *P. frutescens*, 47 syntenic pairs of *PfBAHD* genes were identified. The Ka/Ks ratios for these gene pairs, which serve as indicators of selection pressure (where Ka/Ks > 1 denotes positive selection, <1 indicates purifying selection, and = 1 suggests neutral evolution), varied from 0.02674 to 0.5513 (Ka: 0.00290–0.5685; Ks: 0.02943–2.3221), with all values being less than 1 (Table S4). These findings strongly indicate that the *PfBAHD* gene family has predominantly experienced purifying selection, suggesting significant functional constraints on its protein-coding sequences throughout evolutionary history.

### 3.5. Comprehensive Profiling of Tissue-Specific Secondary Metabolites in P. frutescens

**Figure 6:**
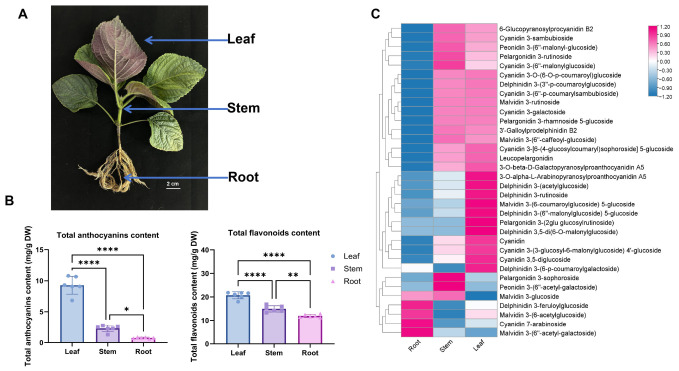
Component analysis of different tissues of *P. frutescens.* (**A**). The different tissues of *P. frutescens*. (**B**). Total anthocyanin accumulation of different tissues and total flavonoid accumulation of different tissues (n = 6); (**C**). Heatmap analysis of anthocyanin-related compounds (n = 3). Note: The **** represents *p* < 0.0001, ** represents *p* < 0.01, * represents *p* < 0.05. Data represent the means ± SDs.

To identify BAHD acyltransferases associated with anthocyanin biosynthesis in *P. frutescens*, we performed a comprehensive metabolic analysis of the roots, stems, and leaves. This analysis included quantification of total flavonoids and anthocyanins, complemented by LC-MS-based untargeted metabolomics. Our findings indicated that the leaves exhibited the highest total anthocyanin content at 9.29 mg/g dry weight (DW), which was significantly greater than the levels found in the stems (2.7 mg/g DW) and roots (0.77 mg/g DW). Similarly, the leaves contained the highest total flavonoid content at 20.72 mg/g DW, compared to 14.93 mg/g DW in the stems and 11.96 mg/g DW in the roots (Figure 6A,B). The observed differences in metabolite content among the tissues were statistically significant (*p* < 0.05), aligning with the purple phenotype characteristic of the leaves and stems in *P. frutescens*.

An untargeted metabolomics analysis identified a total of 3202 secondary metabolites, of which 1545 were common across all three tissues—roots, stems, and leaves. Specifically, 149, 98, and 360 metabolites were uniquely identified in roots, stems, and leaves, respectively (Figure S2). The classification of these metabolites indicated that flavonoids (34.96%), terpenoids (30.49%), and phenolic acids (8.86%) constituted the major components (Figure S3A). Further investigation revealed the presence of 36 anthocyanin compounds, predominantly enriched in stems and leaves (Table S4, Figure 6C). Employing thresholds of *p* < 0.05 and VIP > 1, a total of 1532 differentially accumulated metabolites (DAMs) were identified, comprising 1064 between roots and leaves, 957 between roots and stems, and 707 between stems and leaves, with 108 DAMs common to all three comparison groups (Figure S3B). KEGG enrichment analysis demonstrated that the DAMs were significantly enriched in pathways such as flavone and flavonol biosynthesis, flavonoid biosynthesis, tyrosine metabolism, and phenylalanine metabolism (*p* < 0.05) (Table S5). Furthermore, DAMs identified in the root-versus-leaf and root-versus-stem comparisons exhibited significant enrichment in the monoterpenoid biosynthesis and alpha-linolenic acid metabolism pathways (*p* < 0.05) (Figure S4).

### 3.6. Expression Patterns of Anthocyanin-Related PfBAHDs in P. frutescens

Through functional predictions obtained from phylogenetic analysis, 32 PfBAHD members within the Clade I subfamily—tentatively linked to anthocyanin biosynthesis—were selected for further examination. A correlation analysis, which integrated anthocyanin accumulation levels across various tissues with the expression profiles of these genes, identified that 10 *PfBAHDs* (*PfBAHD112*, *PfBAHD78*, *PfBAHD51*, *PfBAHD73*, *PfBAHD77*, *PfBAHD05*, *PfBAHD126*, *PfBAHD131*, *PfBAHD113*, and *PfBAHD130*) exhibited significantly positive correlations (*p* < 0.05, r > 0.6) with several anthocyanin components, including Cyanidin 3-O-(6-O-p-coumaroyl)glucoside, Cyanidin, and Cyanidin 3,5-diglucoside (Figure S5). These compounds are key intermediates or derivatives in the biosynthetic pathway of shisonin, a distinctive cyanidin-type anthocyanin found in *P. frutescens*, implying that the identified PfBAHDs are likely to function as acyltransferases within this pathway.

**Figure 7:**
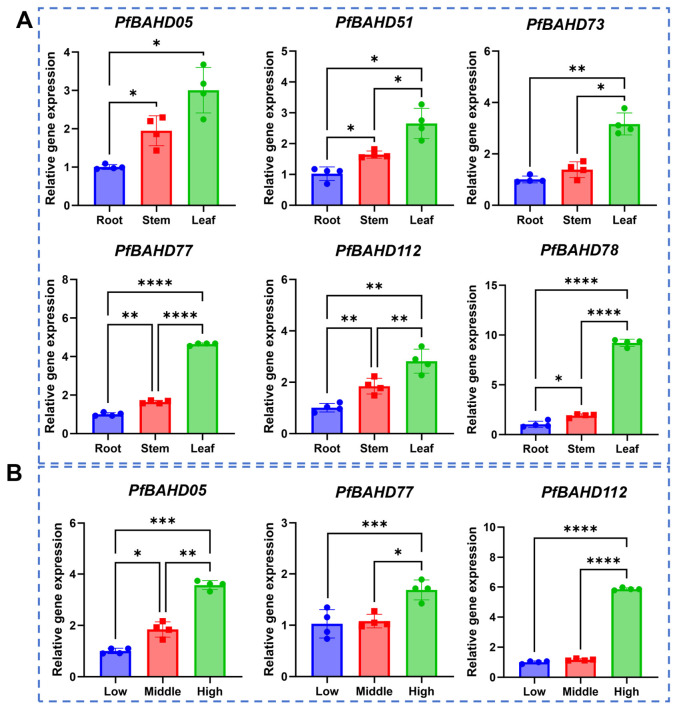
Expression analysis of *PfBAHDs.* (**A**). The qRT-PCR analysis of candidate *PfBAHDs* of different tissues. (**B**). Relative expression levels of *PfBAHD05*, *PfBAHD77*, and *PfBAHD112* under different light intensities (High: 30,000 Lx; Middle: 12,000 Lx; Low: 5000 Lx). Note: The **** represents *p* < 0.0001, *** represents *p* < 0.001, ** represents *p* < 0.01, * represents *p* < 0.05. Data represent the means ± SDs (n = 4).

Further analysis was conducted by identifying the intersection between leaf-specifically highly expressed *PfBAHDs* and Clade I members, resulting in the identification of six candidate genes: *PfBAHD05*, *PfBAHD112*, *PfBAHD77*, *PfBAHD78*, *PfBAHD51*, and *PfBAHD73* (Figure S6). Importantly, the expression levels of all six genes exhibited significant positive correlations with the concentration of cyanidin-type anthocyanins. Validation through qRT-PCR confirmed that their expression was highest in leaves, intermediate in stems, and lowest in roots, aligning with the transcriptomic data (Figure 7A). After comparing with the NCBI database, it was found that *PfBAHD05*, *PfBAHD112*, and *PfBAHD77* are likely anthocyanin-related acyltransferases. Given previous studies highlighting the influence of light on anthocyanin biosynthesis in *P. frutescens* [47,48], we further investigated the expression of these three genes under varying light intensities (low: 5000 lx; medium: 12,000 lx; high: 30,000 lx). The findings revealed that their expression was significantly up-regulated under high light conditions. Under low light conditions, *PfBAHD05* expression was significantly down-regulated, whereas the expression of *PfBAHD77* and *PfBAHD112* remained unchanged (Figure 7B). The light-responsive expression patterns observed further substantiate their potential involvement in the light-regulated anthocyanin biosynthesis pathway, thereby justifying their selection as principal targets for subsequent functional investigations.

### 3.7. Subcellular Localization and Molecular Docking of Candidate PfBAHDs Associated with Anthocyanin Biosynthesis

**Figure 8:**
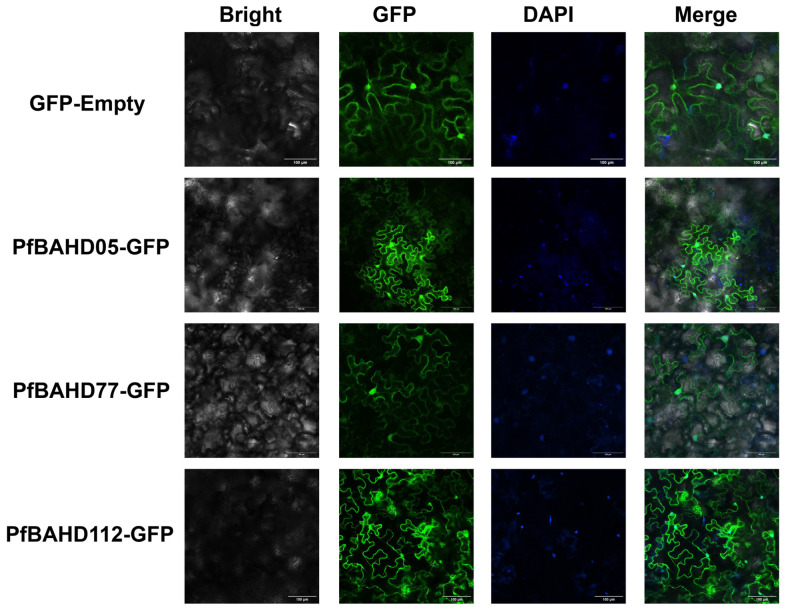
Subcellular localization of PfBAHD05, PfBAHD77 and PfBAHD112 proteins. Selected PfBAHD-GFP fusion protein and GFP-Empty as the control were independently transiently expressed in tobacco leaves and imaged under a confocal microscope. Bars = 100 μm.

To elucidate the subcellular localization of *PfBAHD05*, *PfBAHD112*, and *PfBAHD77*, we engineered expression vectors—pCNG-*PfBAHD05*, pCNG-*PfBAHD77*, and pCNG-*PfBAHD112*—by fusing each respective gene to the N-terminus of the green fluorescent protein (GFP). These constructs were transiently expressed in *Nicotiana benthamiana* leaves via *Agrobacterium tumefaciens*-mediated transformation. Confocal laser scanning microscopy revealed that the fluorescence signals of *PfBAHD05*, *PfBAHD77*, and *PfBAHD112* were predominantly localized within the cytoplasm, corroborating the predicted subcellular localization results (Figure 8).

In order to elucidate the functions of PfBAHD05, PfBAHD112, and PfBAHD77, we constructed their three-dimensional protein models and conducted molecular docking studies with four anthocyanin substrates (Figures S7–S15). The structural models of PfBAHD05 and PfBAHD112 exhibited high similarity, both being based on the anthocyanin acyltransferase template 7dex.1, whereas PfBAHD77 was modeled using the malonyltransferase template Q8W1X0.1. The docking analysis revealed distinct substrate preferences among the proteins. PfBAHD05 and PfBAHD112 demonstrated the strongest binding affinity (Vina scores ranging from −8.7 to −8.3) for the less-glycosylated Cyanidin. Conversely, PfBAHD77 showed a pronounced preference for Cyanidin 3-O-rutinoside, with a Vina score of −9.6. Further analysis of the protein-ligand complexes corroborated this divergence; PfBAHD05 and PfBAHD112 formed more hydrogen bonds with Cyanidin and Cyanidin 3,5-diglucoside, whereas PfBAHD77 established the highest number of bonds (8) with Cyanidin 3-O-rutinoside. These findings suggest that PfBAHD77 may be specialized in recognizing and acylating highly glycosylated anthocyanins, such as Cyanidin 3-O-rutinoside, potentially contributing to late-stage modifications. Conversely, PfBAHD05 and PfBAHD112 likely possess broader substrate adaptability and could be involved in earlier acylation steps of the pathway. In conclusion, we speculate that the aforementioned three PfBAHDs may be associated with anthocyanin acylation in *P. frutescens*.

## 4. Discussion

*P. frutescens* is a valuable herb distinguished by its accumulation of unique acyl-modified anthocyanins, notably shisonin and malonylshisonin [49]. These pigments are highly regarded for their coloration and bioactive properties; however, their practical application is limited by their inherent instability under environmental stress conditions [50]. The process of acylation, which significantly enhances anthocyanin stability through intramolecular copigmentation, is vital for the persistence of these compounds in *P. frutescens* [51,52]. This modification is typically facilitated by BAHD acyltransferases. Consequently, elucidating the BAHD acyltransferase family in *P. frutescens* is crucial for comprehending the molecular basis of its stable and distinctive anthocyanin profile. To date, the BAHD family has been systematically characterized in several plant species, including *Arabidopsis thaliana* (52 members), *Prunus avium* (125 members), *Rubus mesogaeus* (69 members), *Musa acuminata* (46 members), *Panax ginseng* (103 members), and *Camellia sinensis* (112 members) [53,54,55]. In this study, we identified 134 *PfBAHD* members within the *P. frutescens* genome, a quantity exceeding those identified in model plants such as *A. thaliana*. This finding may suggest a potentially broader range of functional roles for this family in the secondary metabolism of *P. frutescens*. Furthermore, the functional diversification of the BAHD family has been demonstrated across multiple species throughout plant evolution, such as *A. thaliana*, *S. lycopersicum* [56,57].

Phylogenetic analysis has categorized the PfBAHDs into six distinct clades, thereby affirming the functional diversity within this family. Notably, Clade I, comprising 32 PfBAHDs, is particularly enriched with enzymes that acylate anthocyanins and flavonoids across various species (e.g., Dv3MaT, Ss5MaT1) [35,54,58]. The association of PfBAHDs with these functionally characterized homologs offers phylogenetic evidence supporting their potential involvement in anthocyanin metabolism. This hypothesis is further supported at the sequence level, as all PfBAHDs exhibit the conserved catalytic (HXXXD) and CoA-binding (DFGWG) motifs, and the majority of Clade I members possess the YFGN motif, which is frequently associated with anthocyanin-related BAHDs [18]. Moreover, promotercis-element analysis revealed a substantial presence of light- and hormone-responsive elements, including motifs responsive to MeJA, within the *PfBAHD* genes. Importantly, candidate genes with high expression levels in the leaves, such as *PfBAHD05*, *PfBAHD73*, *PfBAHD77*, and *PfBAHD112*, were found to contain numerous light- and MeJA-responsive elements in their promoters. This pattern aligns with the well-documented regulation of anthocyanin synthesis by light and phytohormones. This is particularly significant given that leaves serve as the primary sites for anthocyanin accumulation and the perception of biotic and abiotic stresses that trigger JA signaling.

The expression of BAHD acyltransferases involved in anthocyanin acylation is generally coordinated with pigment accumulation, as demonstrated in *C. sinensis*, where the expression of *CsBAHD05* shows a strong correlation with anthocyanin levels [53,59]. Building upon this concept, we integrated transcriptomic and metabolomic data across various tissues of *P. frutescens*. Our findings confirmed that leaves are the primary site of anthocyanin and flavonoid accumulation, aligning with the observed purple phenotype [60]. Through correlation analysis between Clade I gene expression and anthocyanin content, we highlighted several candidate genes, notably *PfBAHD05*, *PfBAHD77*, and *PfBAHD112*, which showed significant positive correlations with cyanidin-type anthocyanins. These candidates exhibited predominant expression in leaves and were significantly upregulated under high-light conditions, consistent with the established light regulation of anthocyanin biosynthesis. The consistency of these findings was further supported by cross-validation using multi-omics data.

To further explore the potential functions of these three candidates, we examined their homologous genes in other species whose functions have been experimentally verified. The homology models of PfBAHD05 and PfBAHD112 were built using the anthocyanin acyltransferase template 7dex.1, which corresponds to Ss5MaT1 from *S. splendens*. Ss5MaT1 has been characterized as an anthocyanin malonyltransferase that catalyzes the transfer of a malonyl group to the 5-glucosyl moiety of anthocyanins [58]. In contrast, PfBAHD77 was modeled using the malonyltransferase template Q8W1X0.1, i.e., Gt5,3′AT from *G. triflora*, which is a verified aromatic acyltransferase capable of transferring aromatic acyl groups to both the 5- and 3′-positions of anthocyanins [61]. The functional characterization of these homologs is consistent with the predicted acyltransferase activities of the *P. frutescens* candidates.

Subcellular localization of PfBAHD05, PfBAHD77, and PfBAHD112 predicted these proteins to be cytoplasmic, aligning with the typical compartmentalization of their acyl-CoA substrates and phylogenetically related, functionally characterized acyltransferases, such as At5MaT [10]. Similarly, research on the barley *BAHD* gene family indicated that the At5MaT-clustered homolog HbBAHD096 is also localized in the cytoplasm, further supporting their possible involvement in cytoplasmic anthocyanin modification [62]. Furthermore, molecular docking studies indicated distinct substrate preferences: PfBAHD05 and PfBAHD112 exhibited higher affinity for less-glycosylated cyanidin, whereas PfBAHD77 demonstrated a preference for Cyanidin 3-O-rutinoside. These results suggest a hypothesis that PfBAHD77 may catalyze later acylation steps on complex glycosides, such as shisonin, while PfBAHD05 and PfBAHD112 might function earlier in the pathway. This provides a possible mechanistic hypothesis for the biosynthesis of *P. frutescens*-specific anthocyanins. Notably, the distinct template assignments—Ss5MaT1 (a malonyltransferase) for PfBAHD05/PfBAHD112 and Gt5,3′AT (an aromatic acyltransferase) for PfBAHD77—are consistent with their differential substrate preferences observed in molecular docking, further supporting the functional predictions, which remain to be experimentally tested.

This study undertook a comprehensive genome-wide analysis of the BAHD acyltransferase family in *P. frutescens*, suggesting three key candidate genes—*PfBAHD05*, *PfBAHD77*, and *PfBAHD112*—may be associated with anthocyanin biosynthesis through multi-omics correlation. Nonetheless, the precise enzymatic roles of these genes necessitate further validation via in vitro assays and functional genetic methodologies. Future research should prioritize such validation and, subsequently, explore their dual potential for metabolic engineering. First, heterologous expression of these *PfBAHDs* in microbial or plant chassis could be deployed to specifically enhance the production of the native, stable acylated cyanidin derivatives of *P. frutescens*, addressing yield limitations. Second, and more prospectively, leveraging the characteristic substrate promiscuity of BAHD enzymes could enable the biosynthesis of novel anthocyanin variants. By supplying alternative acyl-CoA donors (e.g., from the phenylpropanoid or fatty acid pathways) in engineered systems, these genes could be used as tools to expand the chemical diversity and functionality of bio-sourced pigments. Consequently, harnessing these *PfBAHD* genes may represent a viable pathway to address critical challenges in the industrial-scale production of plant anthocyanins, including low yield, chemical instability, and high processing costs, by precisely redesigning the terminal modification steps. Therefore, leveraging these genetic insights for the tailored biosynthesis of high-value anthocyanins offers a potential frontier for sustainable industrial applications, although this remains speculative pending experimental confirmation.

## 5. Conclusions

A systematic, genome-wide identification of the BAHD acyltransferase family was performed in *P. frutescens*, resulting in the characterization of 134 *PfBAHD* genes. These were phylogenetically classified into six clades (I–VI), with Clade I (32 members) enriched in sequences homologous to functionally validated anthocyanin acyltransferases from other species, suggesting their potential involvement in anthocyanin acylation. Promoter analysis revealed abundant light- and phytohormone-responsive cis-elements, indicating regulation by environmental and developmental signals.

Integration of tissue-specific metabolomic and transcriptomic data identified six candidate genes positively correlated with cyanidin-type anthocyanins. Among them, *PfBAHD05*, *PfBAHD77*, and *PfBAHD112* exhibited predominant leaf expression, up-regulation under high light, and cytoplasmic localization. Molecular docking indicated distinct substrate preferences: PfBAHD77 favored highly glycosylated anthocyanins, whereas PfBAHD05 and PfBAHD112 preferred less-glycosylated substrates. These three genes deserve particular attention as high-priority candidates for understanding anthocyanin acylation in *P. frutescens*. Collectively, this work provides a comprehensive genetic framework and offers valuable targets for future research into the mechanisms controlling anthocyanin modification and for metabolic engineering of stable natural pigments.

## Data Availability

The original contributions presented in this study are included in the article/Supplementary Material. Further inquiries can be directed to the corresponding authors.

## Supplementary Materials

The following supporting information can be downloaded at https://www.mdpi.com/article/10.3390/biology15110859/s1. Table S1. Basic information on other BAHD acyltransferases with genetic or biochemical characteristics in the phylogenetic tree. Table S2. The list of primers. Table S3. Information of the PfBAHD genes family in *P. frutescens*. Table S4. The ka/ks values of PfBAHD genes in *P. frutescens*. Table S5. The intensity of anthocyanin components in *P. frutescens*. Table S6. KEGG enrichment of differential compounds in different parts of *P. frutescens* (with significance *p* < 0.05). Figure S1. Multiple sequence alignment of the protein sequences encoded by the PfBAHDs. Figure S2. TIC analysis of LC-MS for different tissues from *P. frutescens* (A); PCA and Venn analysis of roots, stems and leaves (B,C). Figure S3. The metabolites analysis of different tissues from *P. frutescens*. (A) The classify of secondary metabolites; (B) The differentially accumulated metabolites between different groups. Figure S4. The KEGG enrichment analysis of R vs. L, R vs. S, and S vs. L. Figure S5. Correlation analysis between *PfBAHDs* gene expression of Clade I and anthocyanin component content. Figure S6. PfBAHDs gene expression of different tissues from RNA-Seq (n = 3). Figure S7. The electrophoretogram *PfBAHD05*, *PfBAHD112*, and *PfBAHD77*. Figure S8. The molecular docking of PfBAHD05/PfBAHD112 with the substrate Cyanidin. Figure S9. The molecular docking of PfBAHD05/PfBAHD112 with the substrate Cyanidin-3-O-glucoside. Figure S10. The molecular docking of PfBAHD05/PfBAHD112 with the substrate Cyanidin-3,5-glucoside. Figure S11. The molecular docking of PfBAHD05/PfBAHD112 with the substrate Cyanidin-3-O-rutinoside. Figure S12. The molecular docking of PfBAHD77 with the substrate Cyanidin. Figure S13. The molecular docking of PfBAHD77 with the substrate Cyanidin-3-O-glucoside. Figure S14. The molecular docking of PfBAHD77 with the substrate Cyanidin-3,5-glucoside. Figure S15 The molecular docking of PfBAHD77 with the substrate Cyanidin-3-O-rutinoside.
