# Supplementary material for: Genome-Wide Identification and Analysis of BAHD Acyltransferases Involved in Anthocyanin Biosynthesis in Perilla frutescens
Source: Biology (Basel). 2026 May 30;15(11):859. doi: 10.3390/biology15110859 (PMC13255992; doi:10.3390/biology15110859)
Supplement: Supplementary file 1 [file biology-15-00859-s001.zip › supplyment Figures.pdf]

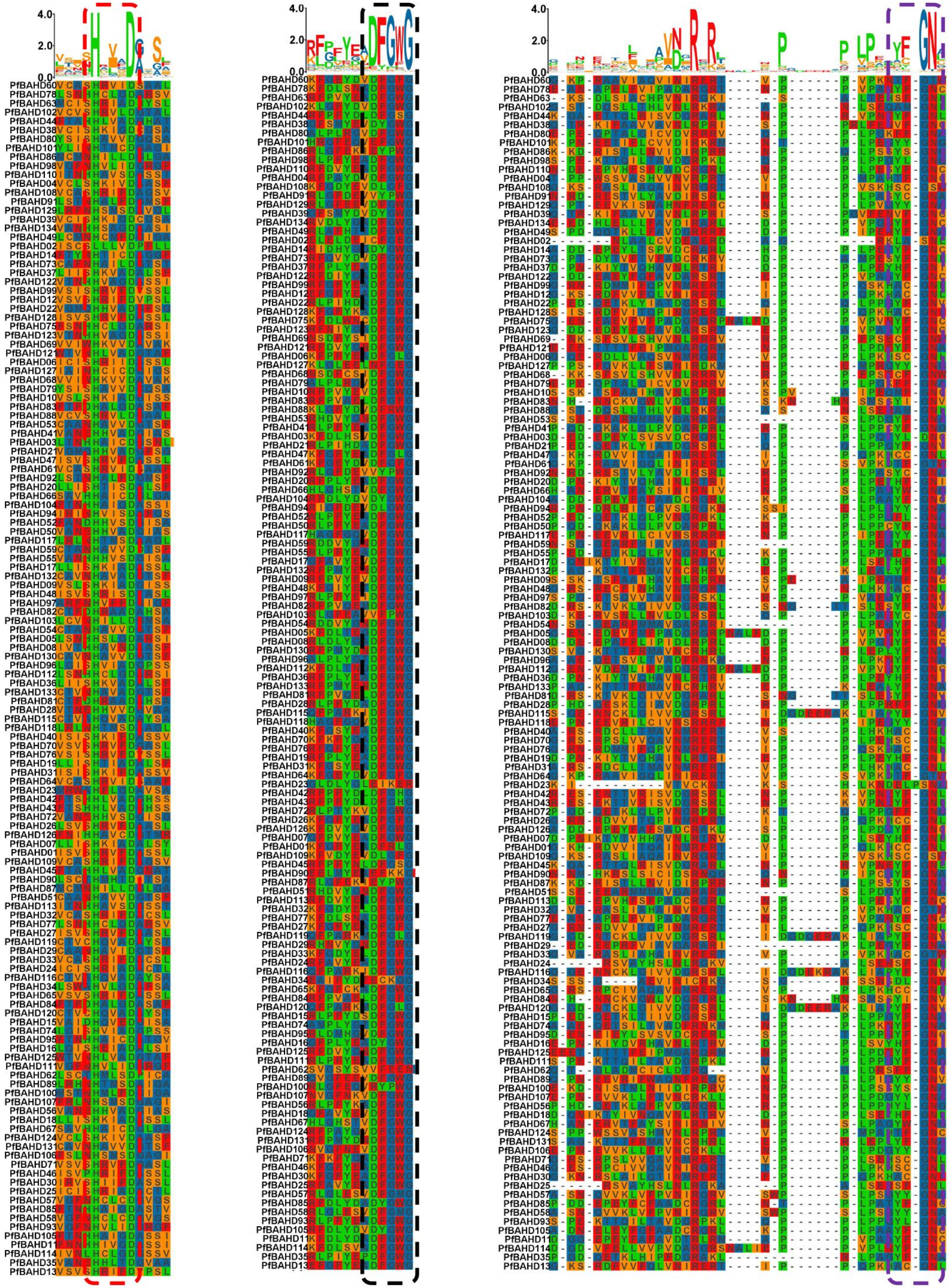

Fig.S1 Multiple sequence alignment of the protein sequences encoded by the PfBAHDs.
Note: The red dashed box identifies the HXXXD domain, the black dashed box identifies the DFGWG domain, and the purple dashed box identifies the YFNG domain..

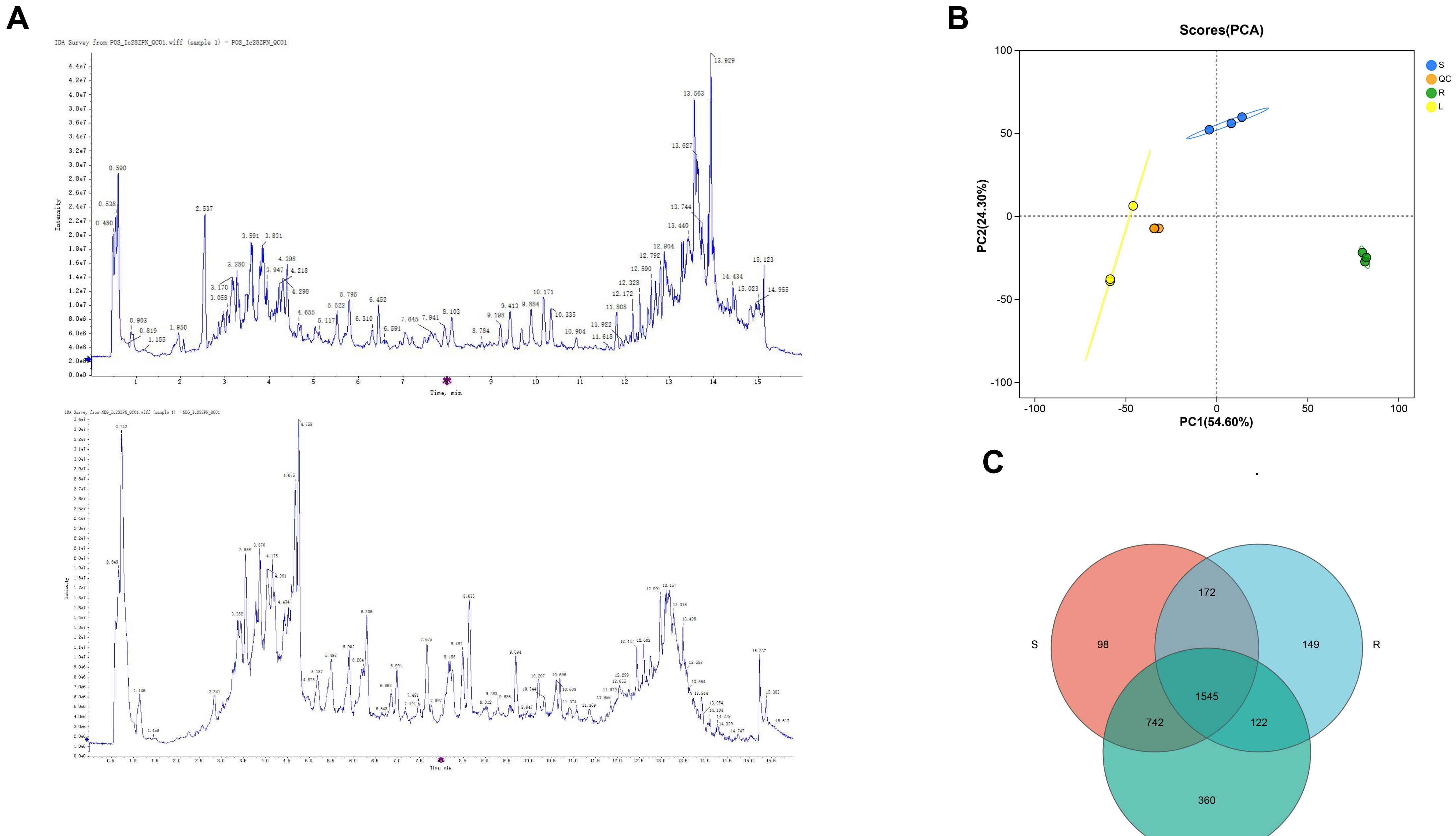

Fig.S2 TIC analysis of LC-MS for different tissues from *P. frutescens* (A); PCA and Venn analysis of roots, stems and leaves (B-C).

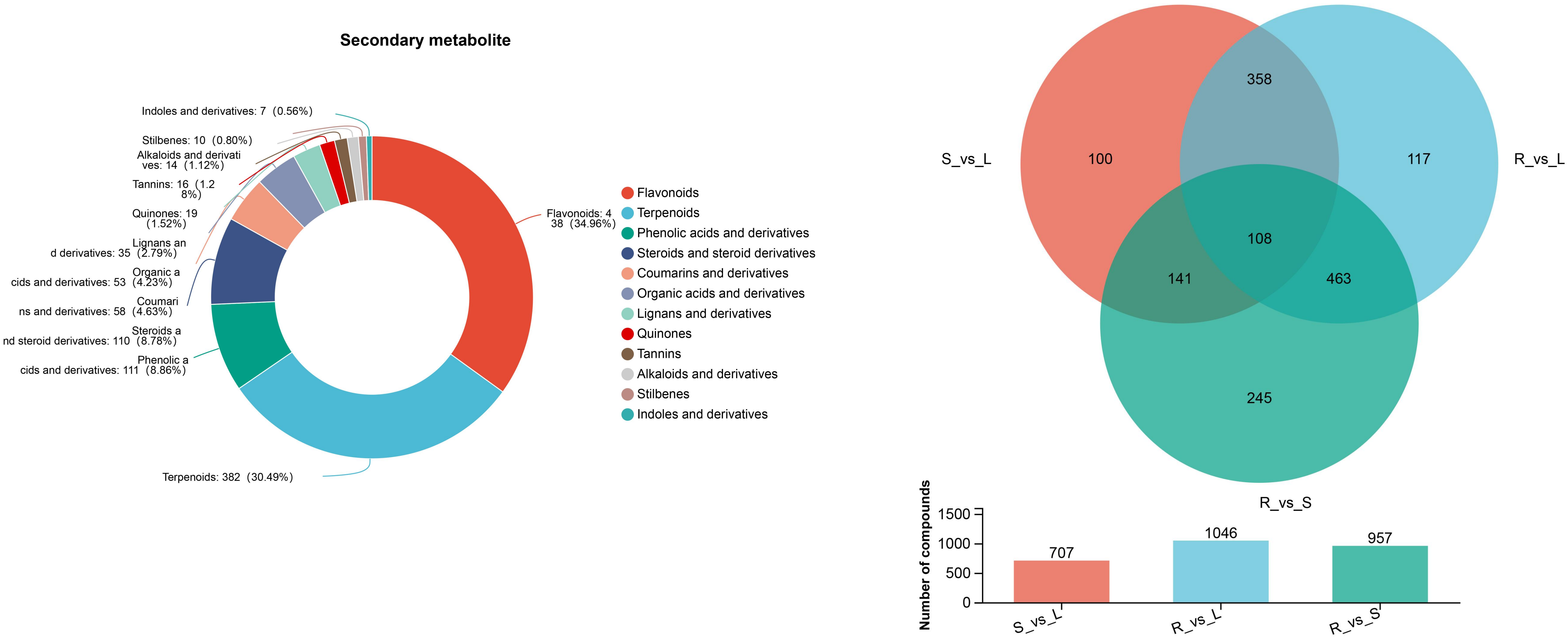

Fig.S3 The metabolites analysis of different tissues from *P. frutescens*. (A) The classify of secondary metabolites; (B) The differentially accumulated metabolites between different groups.

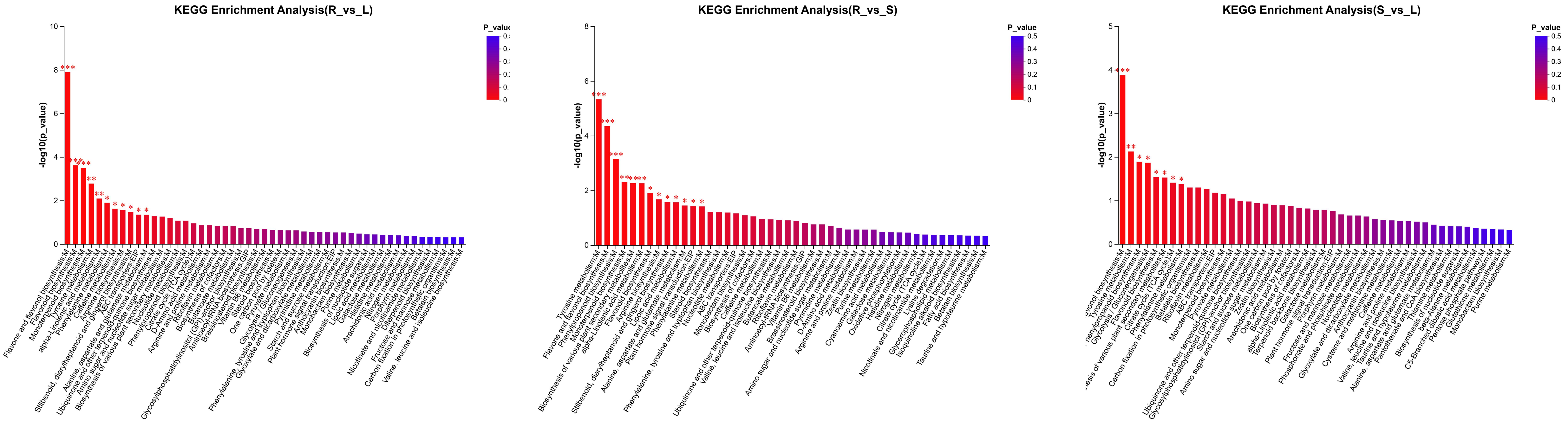

Fig.S4 The KEGG enrichment analysis of R vs L, R vs S, and S vs L.

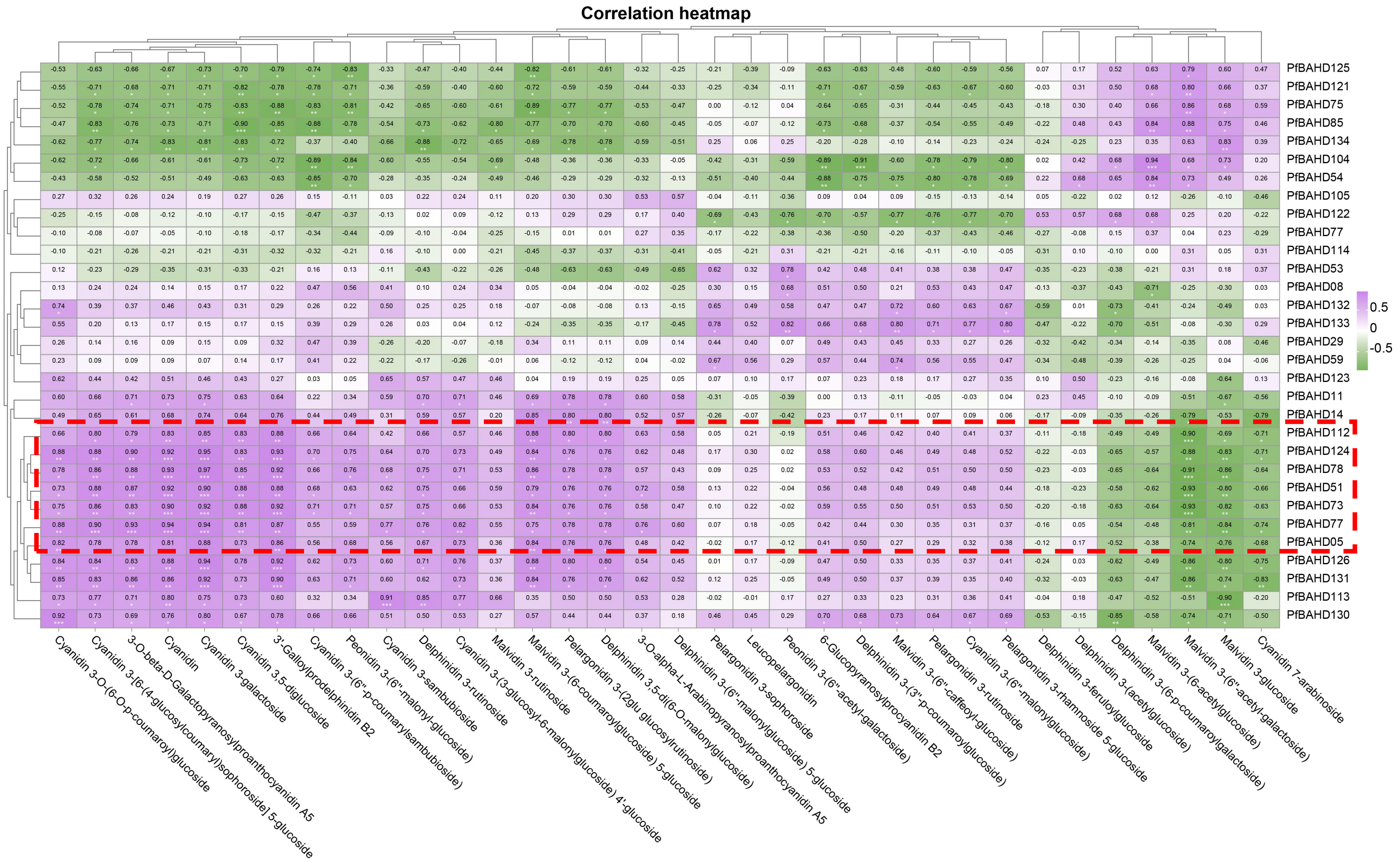

Fig.S5 Correlation analysis between *PfBAHDs* gene expression of Clade I and anthocyanin component content.

Note: The values in the figure represent the correlation coefficient between gene expression and content, *** represents p <0.001, * represents p < 0.05. Data represent the means ± SDs (n=3).

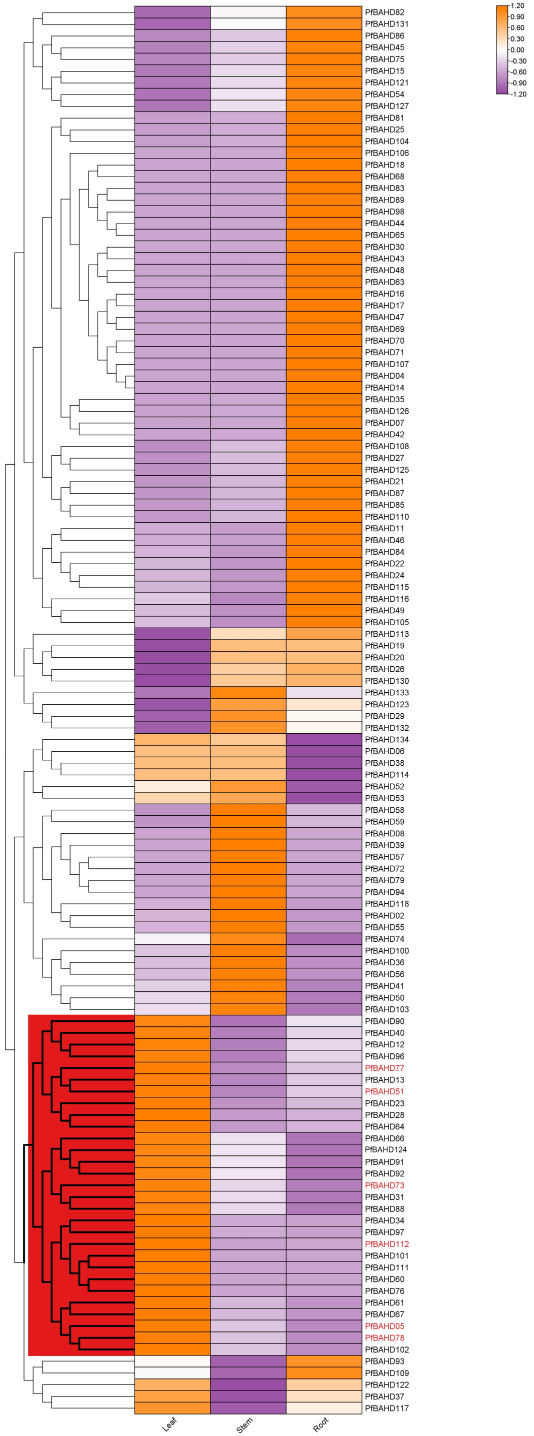

Fig.S6 PfBAHDs gene expression of different tissues from RNA-Seq (n=3). Purple has a low expression level, while orange has a high expression level. The gene expression value is expressed as the count value of log2 conversion. Among them, the genes specifically highly expressed in the leaves are indicated in the clusters in bold red, and the red IDs represent the genes specifically highly expressed in the leaves that intersect with the clade I branch.

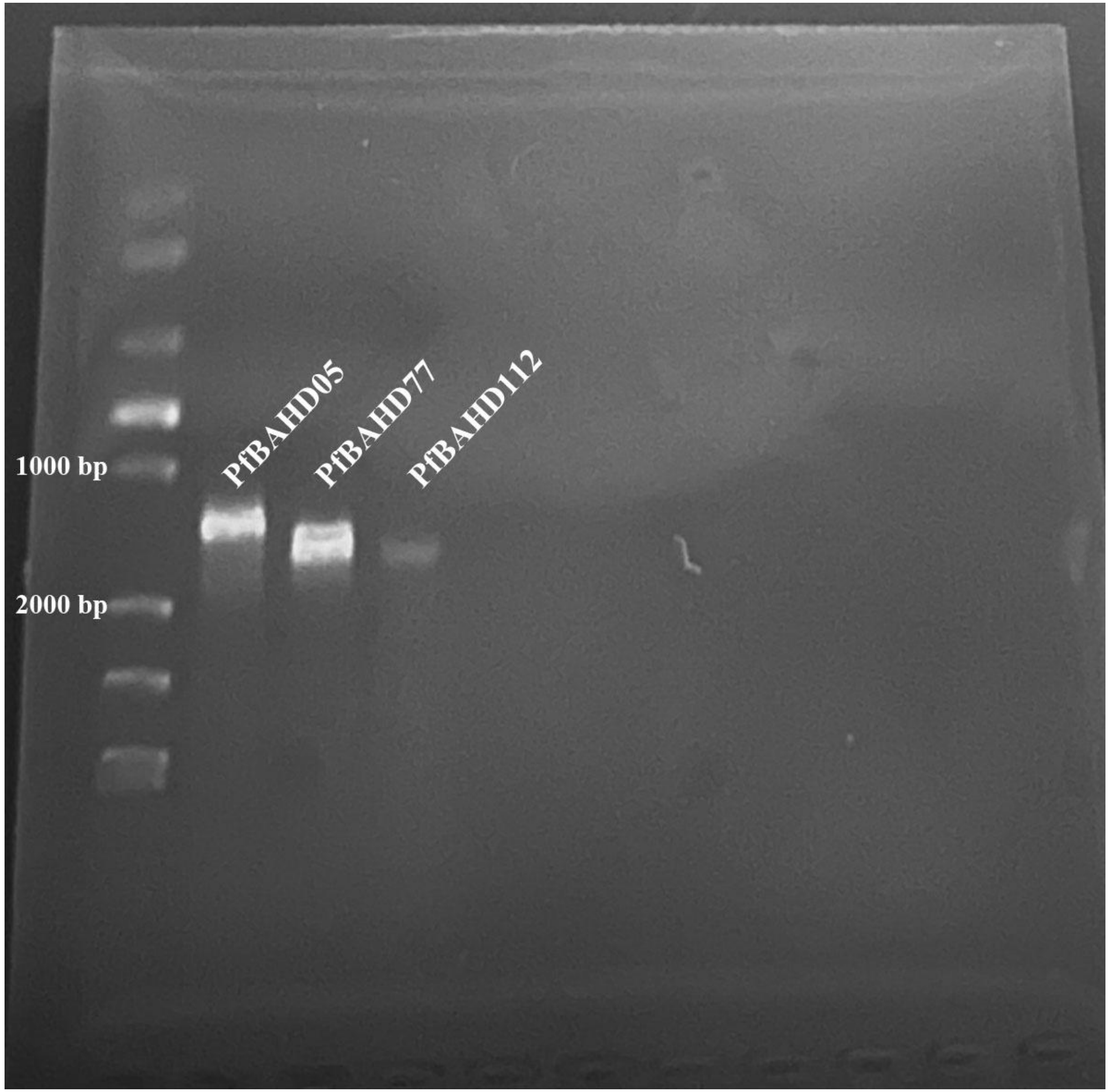

Fig.S7. The electrophoretogram *PfBAHD05*, *PfBAHD112*, and *PfBAHD77*

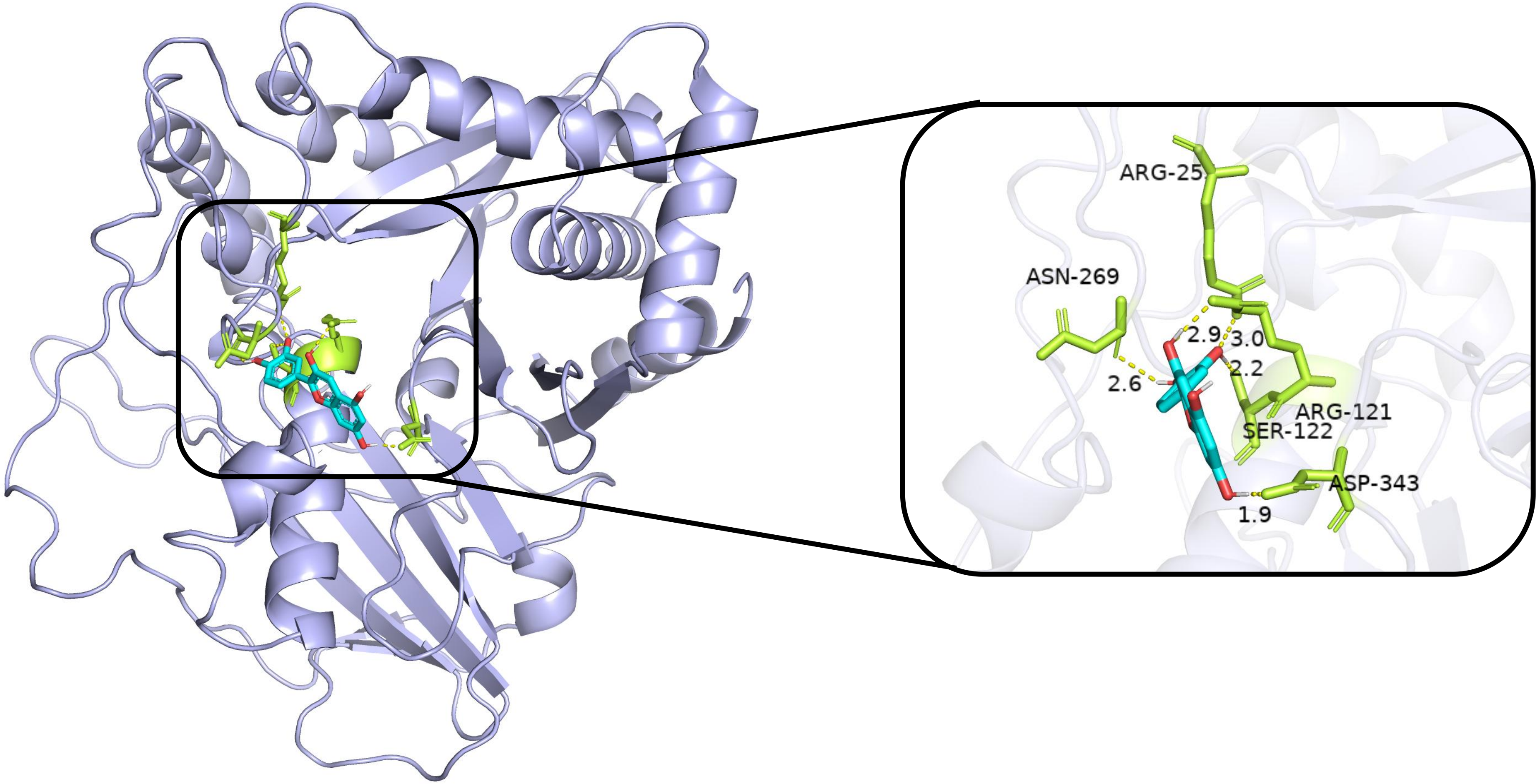

Fig.S8 The molecular docking of PfBAHD05/PfBAHD112 with the substrate Cyanidin.
Note: The yellow color indicates hydrogen bond binding force; the bright purple protein is PfBAHD05/PfBAHD112, the blue small molecule is Cyanidin, and the green color represents the amino acid sites involved in hydrogen bonding.

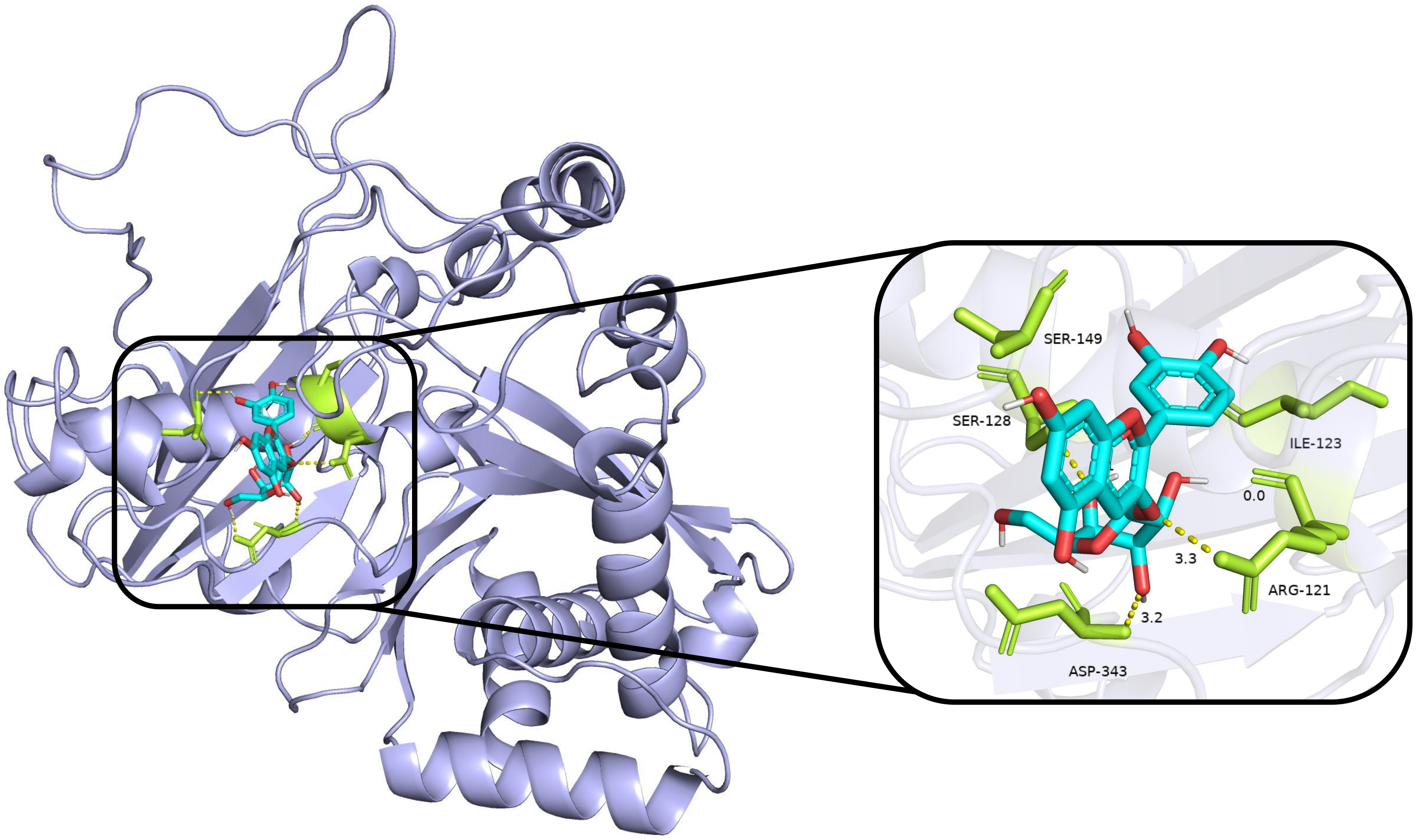

Fig.S9 The molecular docking of PfBAHD05/PfBAHD112 with the substrate Cyanidin-3-O-glucoside.
Note: The yellow color indicates hydrogen bond binding force; the bright purple protein is PfBAHD05/PfBAHD112, the blue small molecule is Cyanidin-3-O-glucoside, and the green color represents the amino acid sites involved in hydrogen bonding.

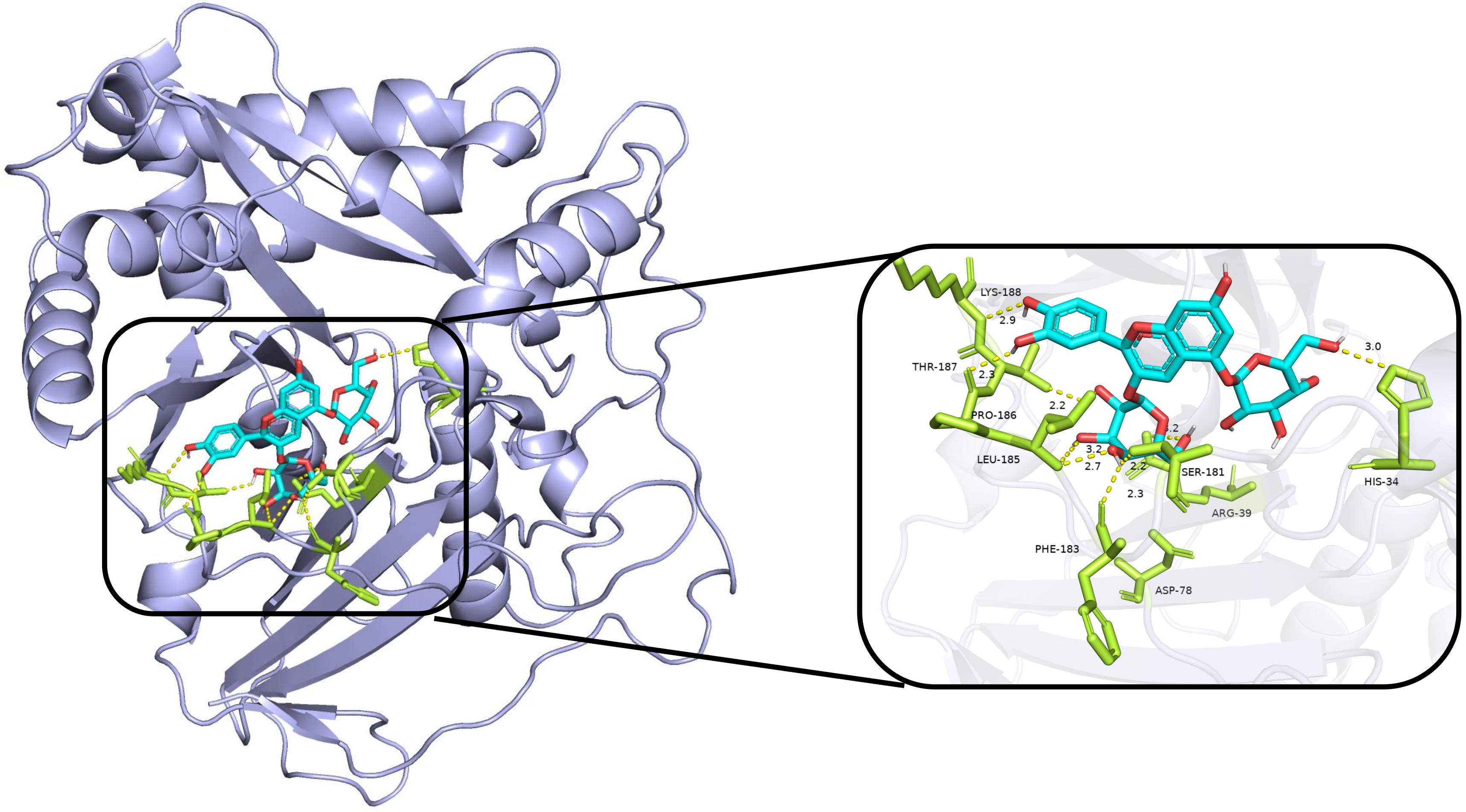

Fig.S10 The molecular docking of PfBAHD05/PfBAHD112 with the substrate Cyanidin-3,5-glucoside.
Note: The yellow color indicates hydrogen bond binding force; the bright purple protein is PfBAHD05/PfBAHD112, the blue small molecule is Cyanidin-3,5-glucoside, and the green color represents the amino acid sites involved in hydrogen bonding.

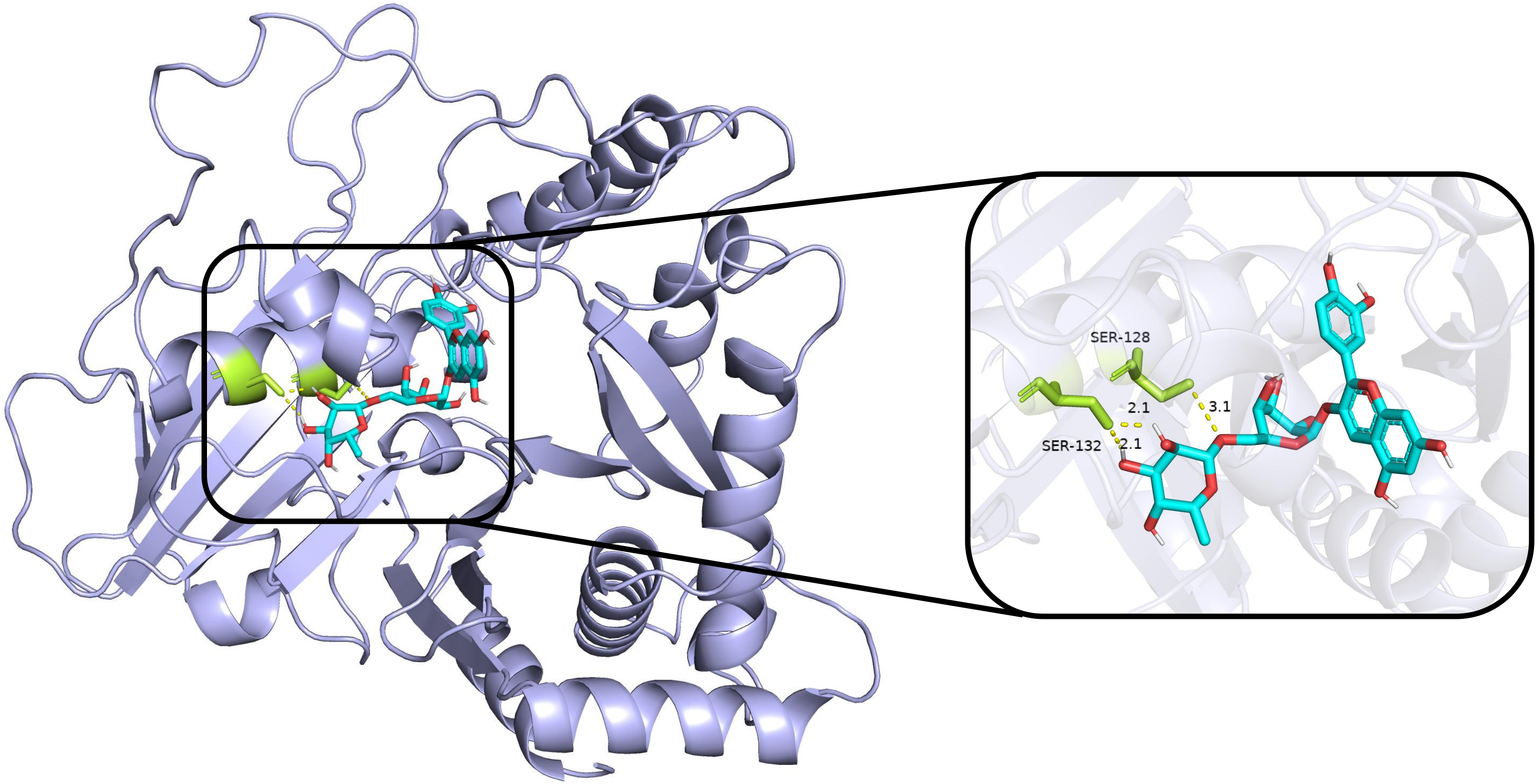

Fig.S11 The molecular docking of PfBAHD05/PfBAHD112 with the substrate Cyanidin-3-O-rutinoside.
Note: The yellow color indicates hydrogen bond binding force; the bright purple protein is PfBAHD05/PfBAHD112, the blue small molecule is Cyanidin-3-O-rutinoside, and the green color represents the amino acid sites involved in hydrogen bonding.

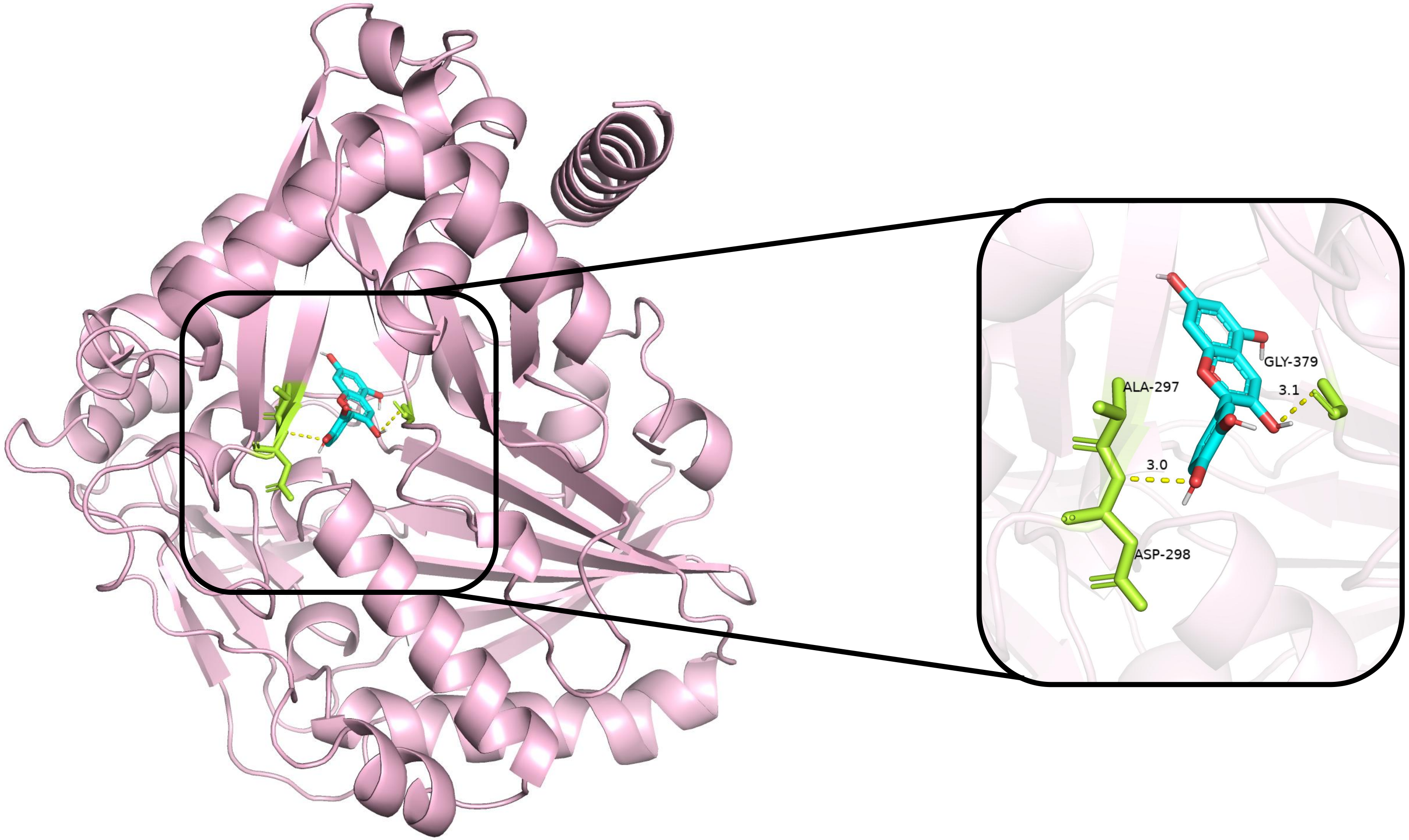

Fig.S12 The molecular docking of PfBAHD77 with the substrate Cyanidin.
Note: The yellow color indicates hydrogen bond binding force; the bright pink protein is PfBAHD77, the blue small molecule is Cyanidin, and the green color represents the amino acid sites involved in hydrogen bonding.

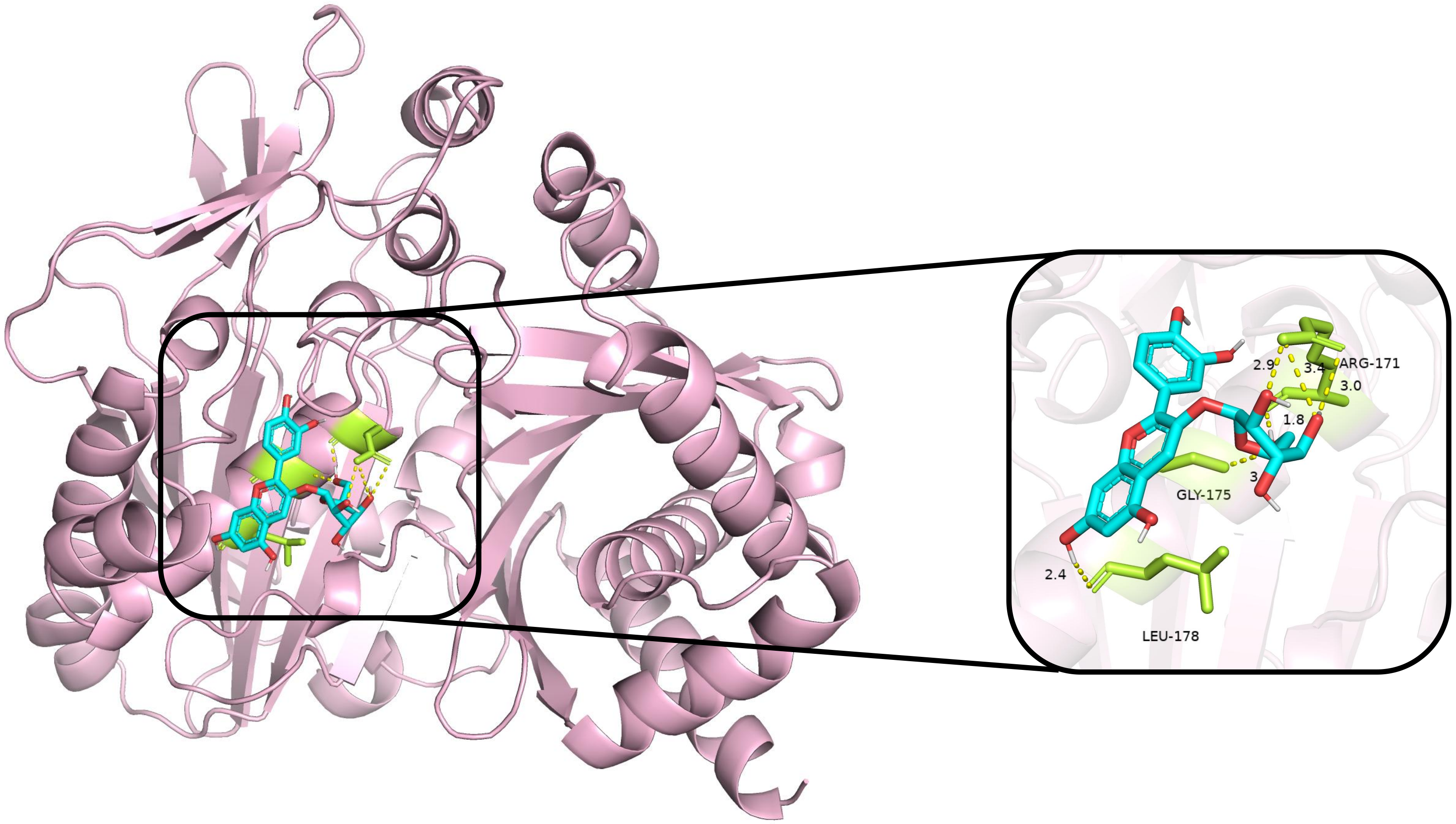

Fig.S13 The molecular docking of PfBAHD77 with the substrate Cyanidin-3-O-glucoside.
Note: The yellow color indicates hydrogen bond binding force; the bright pink protein is PfBAHD77, the blue small molecule is Cyanidin-3-O-glucoside, and the green color represents the amino acid sites involved in hydrogen bonding.

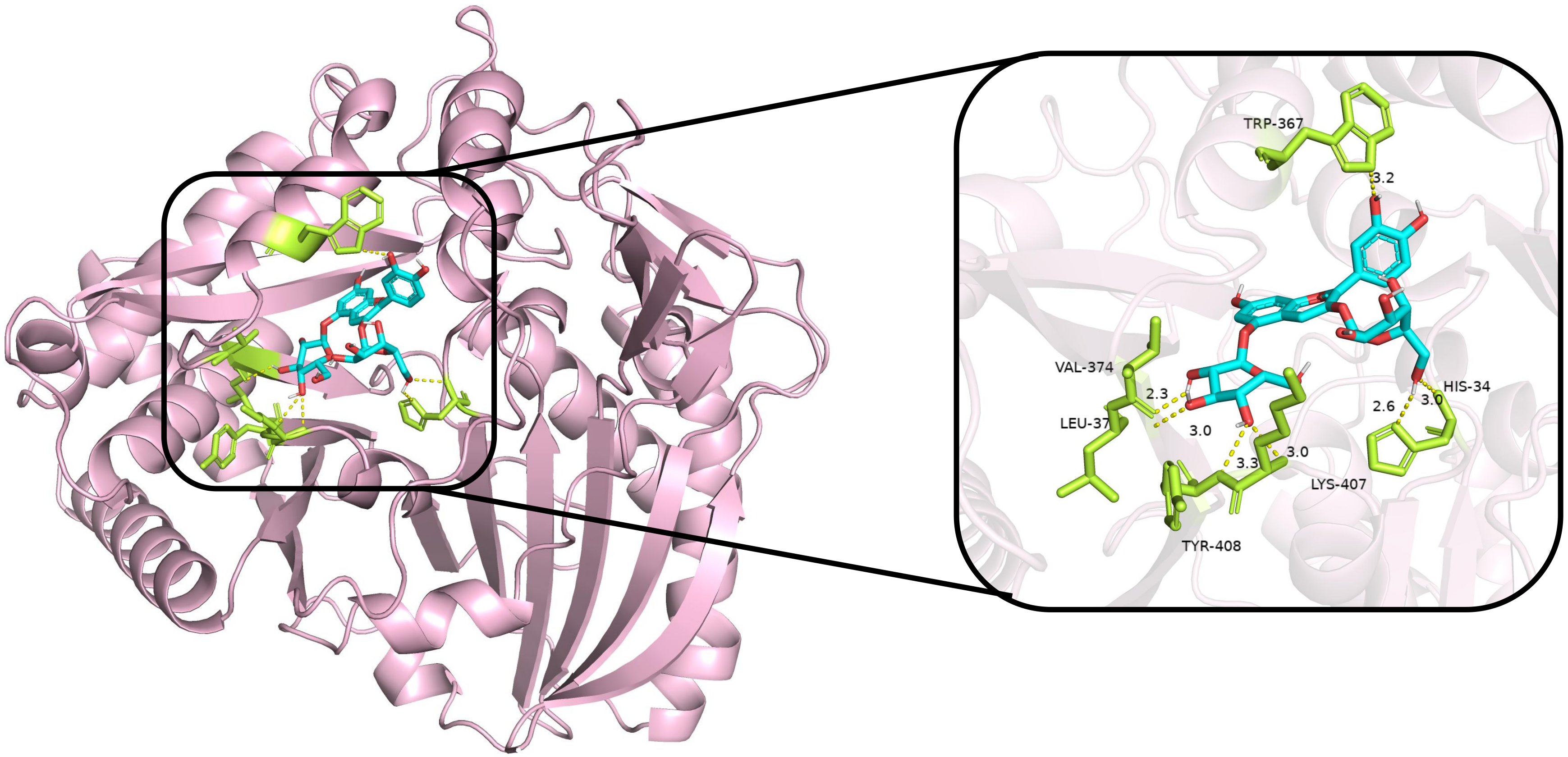

Fig.S14 The molecular docking of PfBAHD77 with the substrate Cyanidin-3,5-glucoside.
Note: The yellow color indicates hydrogen bond binding force; the bright pink protein is PfBAHD77, the blue small molecule is Cyanidin-3,5-glucoside, and the green color represents the amino acid sites involved in hydrogen bonding.

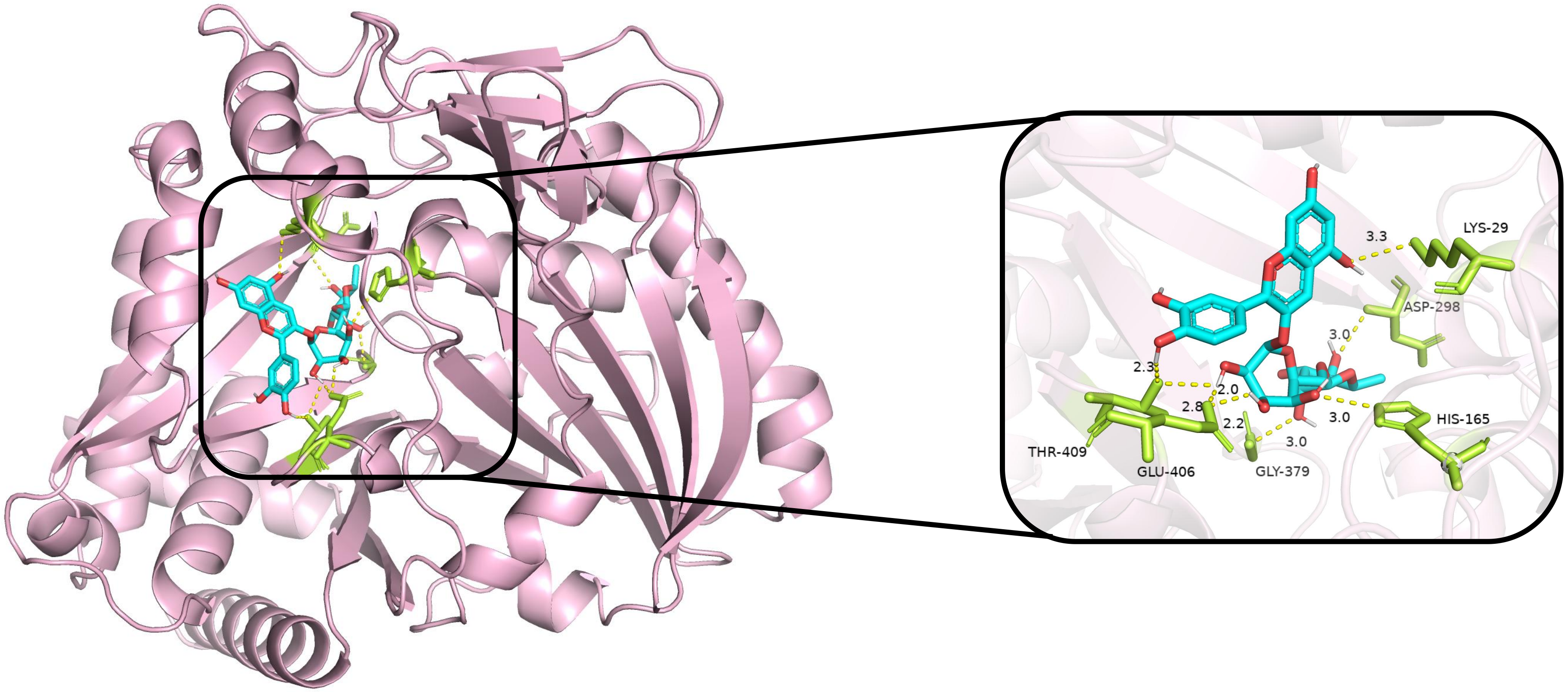

Fig.S15 The molecular docking of PfBAHD77 with the substrate Cyanidin-3-O-rutinoside.
Note: The yellow color indicates hydrogen bond binding force; the bright pink protein is PfBAHD77, the blue small molecule is Cyanidin-3-O-rutinoside, and the green color represents the amino acid sites involved in hydrogen bonding.
